# Few-shot neuromorphic vision in a nonlinear photonic network laser

**DOI:** 10.1126/sciadv.aec6546

**Published:** 2026-09-04

**Authors:** Wai Kit Ng, Jakub Dranczewski, Anna Fischer, T. V. Raziman, Dhruv Saxena, Tobias Farchy, Kilian Stenning, Jonathan Peters, Heinz Schmid, Will R. Branford, Mauricio Barahona, Kirsten Moselund, Riccardo Sapienza, Jack C. Gartside

**Affiliations:** ^1^ Blackett Laboratory, Department of Physics, Imperial College London, London, United Kingdom.; ^2^ IBM Research Europe—Zürich, Säumerstrasse 4, Rüschlikon, 8803, Switzerland.; ^3^ Department of Mathematics, Imperial College London, London, United Kingdom.; ^4^ London Centre for Nanotechnology, Imperial College London, London, United Kingdom.; ^5^ Laboratory of Nano and Quantum Technologies, Paul Scherrer Institut, Switzerland.; ^6^ INPhO, Faculty of Engineering, Ecole Polytechnique Fédérale de Lausanne, Switzerland.; ^7^ Institute for Materials Research, Tohoku University, Japan.

## Abstract

With the growing prevalence of artificial intelligence (AI), demand increases for hardware that mimics the brain’s ability to extract structure from limited data. In the retina, ganglion cells detect features from sparse inputs via lateral inhibition, where neurons antagonistically suppress activity of neighboring cells. Biological neurons exhibit diverse heterogeneous nonlinear responses, linked to robust learning and strong performance in low-data regimes. Here, we introduce a retinally inspired photonic computing system where spatially competing lasing modes in a random network laser act as heterogeneous, inhibitively coupled neurons, enabling feature detection, few-shot classification, and segmentation. This silicon-compatible scheme harnesses heterogeneous excitatory and inhibitory nonlinear physical dynamics, which give rise to emergent photonic computing behavior, including parallel feature detection and strong performance when training data are scarce. We report 98.05 and 87.85% accuracy on MNIST and Fashion-MNIST, and 90.12% on BreakHis cancer diagnosis, outperforming software convolutional neural networks including EfficientNetV2 and the vision transformer ViT in few-shot and class-imbalanced regimes with training sets of up to several hundred images. We demonstrate combined segmentation and classification on the HAM10k skin lesion dataset, achieving DICE and Jaccard scores of 84.49 and 74.80%. These results demonstrate the potential of random lasing networks as nonlinear photonic learning systems and highlight the ability of heterogeneous nonlinear dynamics to support strong learning in challenging low-data scenarios.

## INTRODUCTION

Artificial intelligence (AI) is increasingly ubiquitous across society and industry. Demand grows for dedicated AI hardware capable of strong performance in use cases where training data and energy are scarce, such as edge computing applications where limited data are collected locally and the power and cooling overheads of GPUs are unattractive. These tasks, including image classification, medical diagnosis, and segmentation, require a range of capabilities: (i) strong nonlinearity—crucial for harder tasks (*1*–*3*); (ii) extraction of a range of informative features from input data—neural networks capable of this such as convolutional neural networks (CNNs) (*4*, *5*) and transformers (*6*, *7*) typically outperform those without, e.g., multilayer perceptrons (MLPs) (*4*, *5*, *8*, *9*); and (iii) the ability to rapidly learn from few training examples—termed the few-shot/low-data regime (*10*, *11*).

Physics-based neuromorphic computing systems (*1*, *3*, *12*–*17*) have been explored as candidates for edge-AI hardware (*14*), but integrating the above qualities to achieve software-equivalent performance on harder problems with very limited training data, such as biomedical tasks, remains challenging. Biological systems offer a compelling blueprint. In the retina, ganglion cells perform spatially sensitive nonlinear processing via lateral inhibition (*18*–*21*), where neurons compete to fire while inhibitively suppressing the activity of neighboring cells. This mechanism enables efficient encoding of spatial structure, even under sparse or noisy input, and the ability to detect many features in parallel from multiple ganglion cells (*18*, *22*). The combination of excitatory dynamics (firing in response to input) and inhibitory ones (suppressing activity of nearby coupled neurons) is crucial to the strong performance of biological learning systems, but to date, physics-based neuromorphic schemes typically focus solely on engineering excitatory dynamics. Addressing inhibitory dynamics alongside excitatory dynamics in photonic systems offers an inviting route toward taking neuromorphic performance closer to the biological systems that inspire it. In addition, the diverse heterogeneous nonlinear responses across ensembles of biological neurons have been linked to robust learning (*23*) and creating effective high-dimensional latent spaces that have been shown to give rise to strong few-shot learning in software networks (*11*).

Photonic computing schemes have demonstrated promising routes to high-bandwidth, low-energy performance, with the realization of efficient optical nonlinearities identified as a key area requiring further development (*2*, *24*, *25*). Recent studies have shown that access to strong nonlinearities can substantially enhance computational capability, for example, through optoelectronic nonlinear materials (*26*), nonlinear mode mixing in multimode fibers (*17*), or nonlinear dynamics such as lasing mode competition in multimode vertical-cavity surface-emitting lasers (VCSELs) (*27*, *28*). Exploration of combining heterogeneous nonlinearities is particularly promising here, integrating multiple distinct nonlinear dynamics, which has been shown to give rise to robust learning in software models of biologically inspired neurons (*23*). Developing physical learning systems that exhibit both strong heterogeneous nonlinearities and the capability for useful feature extraction is hence highly attractive, with experimental demonstration of schemes integrating these qualities so-far elusive.

Here, we present a retinally inspired neuromorphic vision system exhibiting strong heterogeneous photonic nonlinearities, parallel feature detection, and strong learning performance in the few-shot/low-data regimes where training examples are scarce. Our system is implemented in a 150-μm-diameter on-chip semiconductor random network laser (*29*), fabricated through the mature process of wafer-bonding indium phosphide (InP) on oxide-coated Si, followed by electron beam lithography and reactive ion etching (*30*).

These complex random lasers host many strongly interacting lasing modes (*31*–*34*), with nonlinear physics well suited for use in a neuromorphic system. The large population of modes is spatially and spectrally varying with complex disordered distributions and provides strong intrinsic heterogeneous photonic nonlinearity, including both excitatory dynamics, where modes reach their threshold and lasing activates, and inhibitory dynamics, where spatially overlapping modes antagonistically compete for finite optical gain (termed “mode competition”) (*35*–*37*). This combination of competing processes mirrors the coupled excitatory and inhibitory dynamics of retinal ganglion cells, where cells attempting to activate in response to incoming light are laterally inhibited by neurotransmitters from neighboring cells. In the retina, this directly gives rise to biological feature and edge detection (*18*–*22*).

The networks’ sensitivity to structured optical input (*34*, *35*, *37*, *38*) makes them an intrinsically well-matched platform for spatial processing, such as images. We demonstrate that the competing excitatory and inhibitory nonlinear photonic dynamics give rise to parallel image feature detection, in addition to the higher-level downstream vision tasks of classification and segmentation, with strong performance in few-shot/low-data regimes. Crucially, the system outperforms a range of software neural networks when training data are scarce, including a pretrained vision transformer (*39*) (ViT_b_16, 86 million parameters) and a pretrained large-scale CNN (*39*) (EfficientNetV2-B0, 7.9 million parameters) on hard biomedical tasks with scarce and class-imbalanced training data.

These results motivate the integration of heterogeneous nonlinear physical dynamics and feature extraction capabilities in physics-based learning systems as a highly promising direction for performing challenging few-shot/low-data learning tasks, crucial to deliver the growing demand for edge-computing systems capable of performing both inference and training (*40*, *41*). Additionally, we show that random network lasers can provide such integrated capability in on-chip photonic systems. By embedding bioinspired behavior combining excitation, inhibition, and nonlinear neuronal heterogeneity directly into the physical dynamics, we enable neuromorphic processing that meets the demands of edge AI.

## RESULTS AND DISCUSSION

### Working principle

**Fig. 1.**
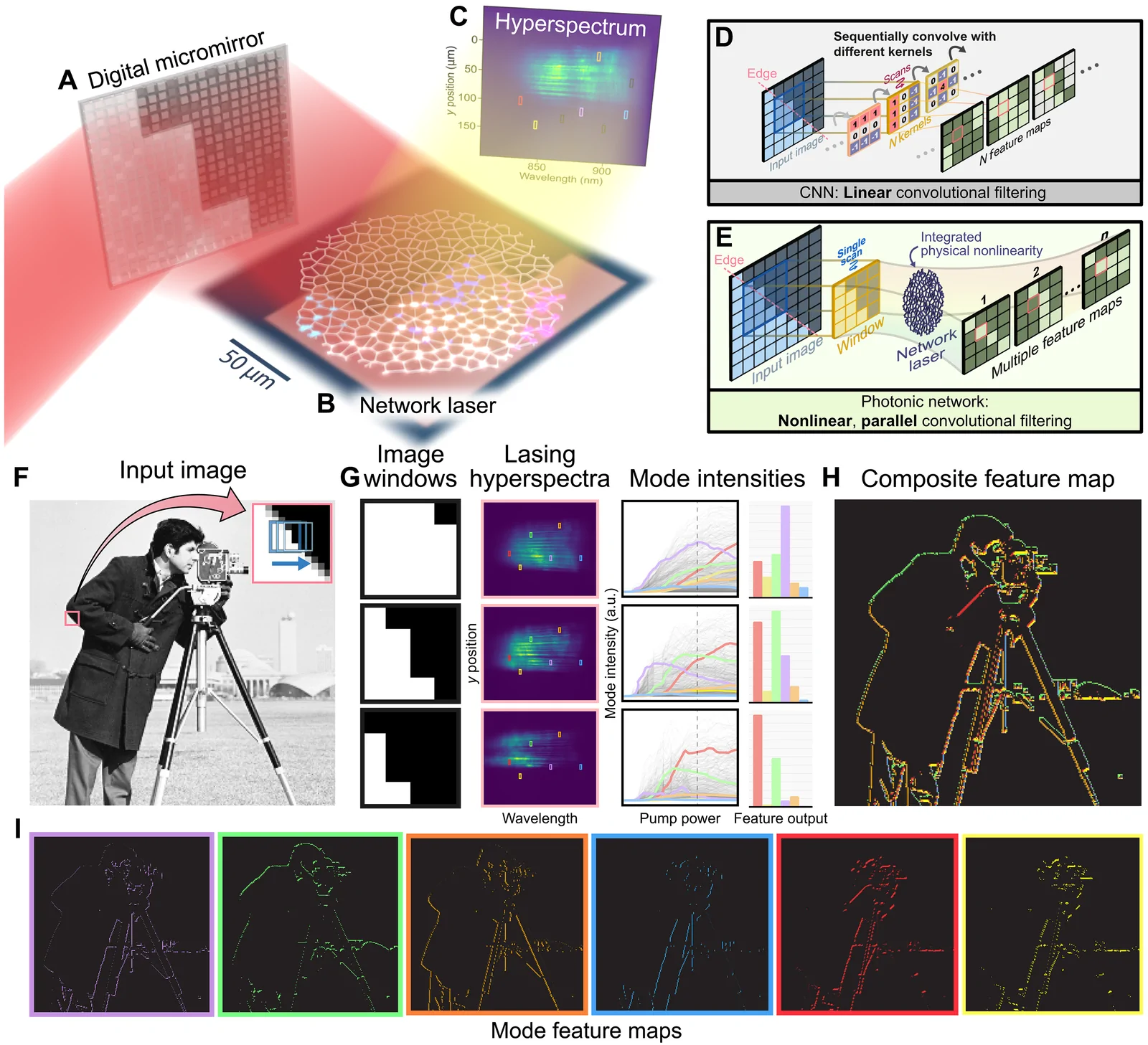
Working principle of network laser vision scheme. (**A**) DMD spatially structures 633-nm, 200-fs pulse pump light into an arbitrary input image, which illuminates a 150-μm InP network (**B**). The pump light generates optical gain in illuminated network regions, exciting photons, which scatter through the network to produce many lasing modes at different wavelengths determined by the scattering paths. Experimentally measured profiles of four lasing modes are indicated via different color network nodes. (**C**) Lasing spectra emitted by the network are recorded via spatially resolved spectrometer, giving “hyperspectral” data with spectral and spatial dimensions, at ∼1 μm spatial resolution. Spectral regions that detect image features are highlighted by colored boxes, corresponding to feature maps in (H) and (I). (**D**) Schematic of feature detection via kernel filters in software CNNs. Linear kernel filters are raster-scanned over an input image to produce feature maps. Each image feature (e.g., left or right edge) is detected sequentially by separate kernel filters. (**E**) Schematic of network laser feature detection. Small input image windows are illuminated on the network in a raster-scan. Different lasing modes detect image features in parallel, with intrinsic physical nonlinearity. (**F**) Input “Cameraman” image; inset shows example 4 × 4 image windows (blue boxes) and raster-scan direction. (**G**) Image windows from (F) with corresponding network spectra (second col.), LL plots showing the heterogeneous nonlinear response of network modes in response to different input images (third col.), and histograms showing mode intensities for six feature-detecting lasing modes (fourth col.). Mode amplitudes vary in response to the presence of different image features, enabling feature detection. (**H**) Composite feature map detected by the InP network. Different colors correspond to the lasing amplitude of the six modes. (**I**) Separate feature maps from the lasing modes, which form the composite map (H). Cameraman image used under non-CC TOU license from MIT https://dome.mit.edu/bitstream/handle/1721.3/195767/cameraman_non_cc_tou.txt. a.u., arbitrary unit.

Our physical system is a 150-μm-diameter InP network laser comprising interconnected lateral nanoscale waveguides, lithographically patterned and etched from a silicon (Si)–bonded InP layer (*29*, *30*). The network has a random Voronoi topology with three waveguides meeting at each vertex (average waveguide length = 5 μm). Fabrication and optical setup details are provided in Materials and Methods and fig. S1. A pump beam, spatially structured via digital micromirror device (DMD) (Fig. 1A), induces optical gain in the InP network (Fig. 1B). Photons are generated by spontaneous emission and scatter through the waveguides, amplified by stimulated emission from the illuminated InP. Constructive interference along specific scattering paths defines optical modes, with the vast number of potential scattering paths giving rise to a large number of spatially distributed distinct overlapping lasing modes, which nonlinearly compete for gain. The modes undergo net gain or loss depending on the extent of overlap between the illumination pattern and the mode profiles. The ∼100-nm bandwidth of the lasing spectra is given by the gain spectrum of the network material (InP), roughly centered around the bandgap.

Pump illumination is supplied by a 633-nm, 200-fs pulse laser, with pulse energy measured at 43 nJ at the sample (under full illumination). The spatial distribution of light emitted from the network by three different modes is illustrated by the colored network nodes in Fig. 1B), experimentally imaged by a frequency-resolved slit-scan camera. The network lasing response is measured using a two-dimensional (2D) charge-coupled device (CCD) detector with diffraction grating. This allows collection of 2D hyperspectra (Fig. 1C) with one spectral dimension, and one spatial dimension at ∼1 μm spatial resolution, and each hyperspectrum is acquired by a single camera image acquisition. The broad lasing modes in the spectral dimension originate from band-filling and rapid carrier-induced index changes caused by ultrashort pulse excitation. Colored boxes on the hyperspectrum correspond to the location of six feature-detecting modes examined in Fig. 1 (F to I) (additional details in fig. S2).

Figure 1D shows a schematic of feature detection in conventional software CNNs. A kernel filter is raster-scanned across small image “windows” of an input image. The kernel values are matrix multiplied against image pixel values, and the results were summed to a single number, which becomes the pixel value of an output feature map, centered at the image window location. This process is repeated across the input image to build feature maps, with different kernel filters sensitive to different image features, e.g., left or right edges. To detect multiple image features, the process is repeated sequentially using different kernels, consuming time and energy. This process is entirely linear, with subsequent nonlinear activations performed at time and energy cost in order to use the feature maps for higher-level AI tasks such as image classification or segmentation. This linear matrix multiplication approach is what has so-far been used in physical convolutional feature detection schemes (*42*–*45*). Table 6.

Figure 1E shows a schematic of the network laser feature detection. Image windows are illuminated on the network in a raster-scan fashion similar to CNNs, with the hyperspectral channels of the network’s lasing emission acting as the output. While input parallelization through input wavelength multiplexing has been achieved in photonic schemes (*42*), we achieve feature detection parallelization via the output spectral channels, where different modes at different wavelengths and spatial positions provide sensitivity to different image features. One raster-scan produces multiple feature maps, with integrated physical nonlinearity, 10 experimentally and 40 in simulation. Our network occupies a compact 150-μm footprint on chip, alongside other off-chip optical components (e.g., laser source, DMD, and camera; see the Supplementary Materials).

Figure 1 (F to I) shows experimental parallel feature detection results for a “Cameraman” test image (F). Example image windows [4 × 4 pixels, inset of (F)] are shown in (G) alongside their respective lasing hyperspectra (second col.), light-in versus light-out (LL) plots (third col., network lasing mode intensity versus pump intensity), and a histogram of mode intensities (fourth col.) for six feature-detecting modes, taken at the pump power indicated by dashed vertical lines in the LL plots.

Mode lasing intensity is increased or inhibited by the presence of different image features, due to a combination of correlation between the input image shape and spatial mode profile, and nonlinear mode competition between spatially overlapping modes. This above-threshold mode competition is intrinsic to multimode lasers. As overlapping modes compete for gain, they inhibit each other by spatial and spectral “hole burning” effects (*46*, *47*). These effects can be modeled using steady-state ab initio laser theory (SALT) (*48*), which we have previously used to build a graph-based theoretical framework of network lasers, termed “netSALT” (*34*). The large variety of spatially and spectrally varying modes in our random network laser gives rise to strong mode competition and high sensitivity to pump patterns (e.g., input images) (*29*, *34*). The resulting increase or inhibition of lasing modes with increasing pump power is clearly seen in the LL plots in Fig. 1G, which show strongly heterogeneous nonlinear responses for each mode, which vary substantially depending on the input image.

This combination of excitatory and inhibitory nonlinear responses to input images enables feature maps to be obtained by plotting the lasing amplitude of different modes above the uniform illumination level at different positions in the raster-scan. The mode intensities plotted in the histogram in (G) are used to generate the pixel amplitudes of our feature maps. Figure 1H shows a composite feature map produced by six lasing modes, with different colors corresponding to the lasing amplitude of six modes indicated in Fig. 1 (C and G to I). Figure 1I shows separate feature maps for the modes comprising the composite map. See Materials and Methods for raster-scan details. Feature maps for all 10 feature-detecting modes are shown in fig. S11.

### Parallel feature detection via network lasing modes

**Fig. 2.**
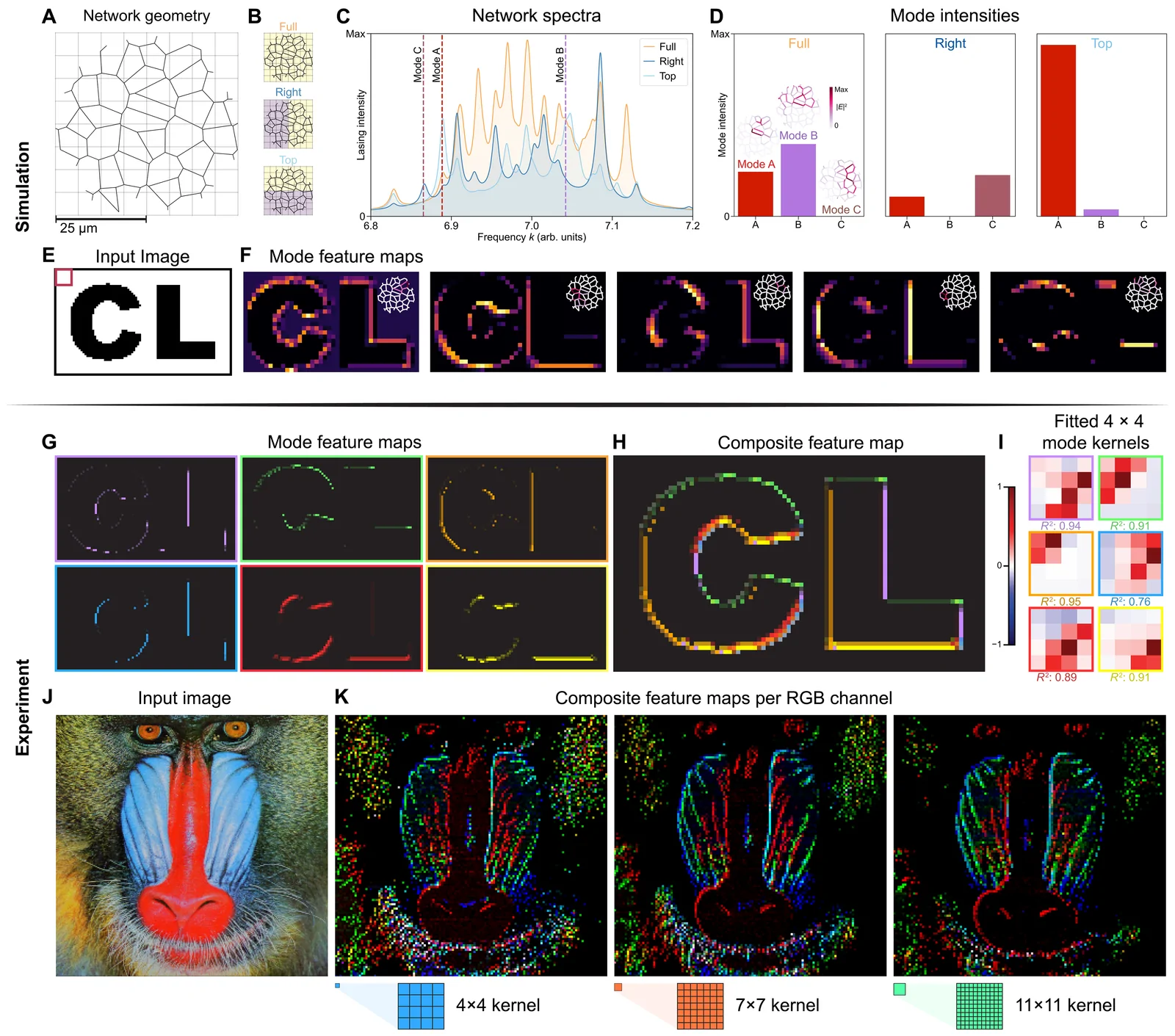
Parallel image feature detection via network lasing modes. (A) to (F) show simulation results, and (G) to (K) are experimental. (**A**) 50-μm network topology used in netSALT simulations (A to F). (**B**) Three pump illumination patterns: uniform, horizontal edge (“right”), and vertical edge (“top”). (**C**) Simulated network lasing spectra for the three illumination patterns. Three modes A to C are indicated. (**D**) Simulated lasing intensities of modes A to C, for the three illumination patterns. Mode competition nonlinearly suppresses specific modes below lasing threshold and amplifies other modes dependent on input pump image. Spatial mode profiles are shown inset in left panel. (**E**) Input image, “CL” for “Complex Lasing.” The input image is fractured into 8 × 8 pixel windows and illuminated onto the network sequentially in a raster-scan, similar to kernel scanning in a convolutional filter process. (**F**) Simulated feature maps for five modes. Pixel brightness in the feature maps corresponds to mode lasing intensity for that position in the raster-scan. Different modes lase in response to different image features; spatial mode profiles shown as insets. A larger set of 28 simulated feature maps and corresponding mode profiles is shown in the Supplementary Materials. (**G**) Experimentally measured “CL” feature maps from six modes. Modes detect specific image features. (**H**) Experimental composite feature map combining modes from (G). (**I**) Equivalent 4 × 4 kernels for the six lasing modes in (G) and (H), extracted by linear fits to experimental data. Colored frames correspond to mode feature map color scheme. (**J** and **K**) Color photograph of a mandrill. Image is separated into RGB channels, and feature detection is performed at multiple kernel sizes demonstrating the resolution flexibility of our scheme. Composite RGB feature maps are shown in (K) for 4 × 4, 7 × 7, and 11 × 11 kernels illustrating the range of high-to-low spatial frequency features detected as kernel size is increased. Kernel sizes are shown below as pixel grids. Mandrill image from USC-SIPI Image Database, reproduced under license from T. Angermayer/*Science Photo Library*.

We can use simulations of the photonic physics to examine the origin of the feature detection. Here, we use netSALT (*34*), a numerical model that solves the nonlinear interaction of optical waves on graph networks within the SALT (*48*) approximation. The netSALT model includes amplification and loss on graph edges and nonlinear competition between spatially overlapping lasing modes. Figure 2A shows a random Voronoi graph network used for the netSALT simulations. Network topology and average edge length (5 μm) match the experimentally measured network shown in Fig. 1, with a smaller 50 μm diameter used in simulation to reduce computational load. Due to the smaller network size (total edge length), the simulated network will have fewer modes than the experimental one.

Three illumination patterns are explored [Fig. 2B: uniform illumination (“full”), horizontal edge (“right”), and vertical edge (“top”)]. The resultant simulated lasing spectra are shown in Fig. 2C, with strong variation in lasing spectra under the different illumination patterns. NetSALT predicts 239 possible lasing modes for the network considered here, with 172 reaching lasing threshold for at least one of the illumination patterns. The three spectra in Fig. 2C show the sum of all active modes for the different illumination patterns. We focus on three specific modes, labeled A, B, and C, identified in Fig. 2C by dashed vertical lines. Figure 2D shows the lasing amplitude of the three modes under “full,” “right,” and “top” illumination patterns, with the spatial mode profiles inset in the left-hand panel. The modes are spatially distributed across the network and overlap considerably. As multiple modes are active in the same network waveguides, nonlinear mode competition occurs between the modes as they antagonistically compete for finite pump gain. Under full illumination, mode B shows the highest amplitude, while mode C does not lase as it falls below threshold. For a right-edge illumination, mode B now falls below the lasing threshold and is deactivated, allowing mode C to lase with the highest amplitude as it no longer faces strong mode competition from mode B. For a top-edge illumination, mode A exhibits far higher amplitude and mode C now falls below the lasing threshold.

Modes A and C exhibit higher lasing amplitude under “top” and “right” edge illuminations, where the network receives less input pump energy than under uniform illumination. This is not possible in a linear system with noninteracting modes and occurs here as removing pump light from some network regions causes modes residing even partially in those regions to lose the mode-competition interactions, e.g., mode B wins under full illumination and loses under edge illumination. This observed behavior shows that nonlinear competition for gain between spatially overlapping modes is providing feature-sensitive lasing dynamics, analogous to the lateral inhibition processes in retinal ganglion cells.

We now consider the netSALT-simulated feature-detection performance of the system. An input image of the letters CL (“Complex Lasing”) is shown in Fig. 2E. The image was fractured into 8 × 8 pixel windows and illuminated on the network in a raster-scan fashion. The lasing response was simulated for each window illumination, and the simulated mode lasing amplitude was plotted at each position in the raster scan to generate separate feature maps for each mode. Figure 2F shows feature maps provided by five different modes, with the corresponding spatial mode profiles inset. These plots demonstrate the ability of the network to perform spectrally multiplexed feature detection of a range of image features, with the broad set of lasing modes effectively providing parallel convolutional filters. The spatial locations of the mode profiles within the network are seen to show correspondence with the detected image features, e.g., modes detecting lower image edges are located toward the bottom of the network (second panel), modes detecting left edges are located toward the left of the network (fourth panel), and so on. Of the 172 active modes in the simulated network, 40 were identified by inspection as exhibiting good feature detection. A set of 28 selected feature maps and corresponding spatial mode profiles is shown in fig. S4, alongside the full set of 172 feature maps from all modes in fig. S5, alongside the simulated mode lasing intensities in fig. S6. A regular hexagonal network was simulated to compare against the random topology (fig. S7), with far fewer detected features in the hexagonal network. This demonstrates the ability of network topology to control the device functionality, with further explorations into the correspondence between topology and performance providing fruitful ground for future work.

We now examine experimental feature detection. Figure 2G shows six experimentally measured feature maps of the “CL” image with colors corresponding to the modes shown in Fig. 1 and a composite feature map shown in Fig. 2H. Parallel feature detection of multiple image features is observed experimentally, with close correspondence to the netSALT-simulated behavior. Figures S8 to S10 examine the feature detection performed by 10 lasing modes across a range of test images. To compare our physically detected feature maps against linear matrix kernel filters as used in software CNNs, we extract effective kernel filters for our modes via least-squares fitting between input images and the experimentally measured mode intensity (details in the Supplementary Materials). The extracted effective filters are shown in Fig. 2I with further details in fig. S20. Notably, both positive and negative kernel weights are observed, as generating bipolar weights is highly challenging in neuromorphic hardware. This is an attractive benefit of the photonic lateral inhibition processes in our system, which enable mode amplitudes to both increase and decrease in response to specific image features. The *R*^2^ values below each effective kernel indicate how well the linear fitting process captures our experimental results. When performing the same kernel fitting process on arbitrary linear software kernel filters, the process performs perfectly with *R*^2^ values of 1.0. The kernels extracted from our experimental data have *R*^2^ values from 0.628 to 0.946 (see figs. S20 and S21), suggesting that aspects of the physical feature detection process cannot be fully captured by linear kernel filters. This is further supported by a similar linear-fit study on netSALT-simulated data shown in fig. S22. The complexity of the fitted kernels also indicates that while these modes were chosen by inspection to produce edge-detecting feature maps, the underlying dynamics are likely to support other convolutional filters.

We extend experimental demonstration of feature detection to high-resolution color images, showing a flexible range of kernel sizes using the popular “mandrill” test image (256 × 246 pixels, not a baboon as often thought) (*49*, *50*). Figure 2J shows the input image, which is separated into RGB channels and illuminated onto the network at a range of kernel sizes to demonstrate the capability of our system to detect features with arbitrary kernel window size. This shows a flexibility of our system in handling input images and kernel windows of varying resolution.

Figure 2K shows experimentally detected feature maps for 4 × 4, 7 × 7 and 11 × 11 kernels, with the R/G/B colored features detected from the corresponding color channel (the response of all 10 feature-detecting modes are summed to produce a single feature map for each color channel here; the separate mode maps can be seen in figs. S17 to S19). The ability to tune feature detection sensitivity to higher or lower spatial frequency is shown in our feature maps produced by different kernel sizes; the 4 × 4 kernel picks up high-frequency details in the mandrill fur around image corners, whereas the 11 × 11 kernel exhibits weaker response to these. Increasingly, powerful modern software CNNs such as EfficientNet (*51*, *52*) use a range of filter sizes, demonstrating the utility and attraction of our ability to freely select filter size. Separate lasing mode feature maps are shown for a wider range of test images (“Cameraman,” CL letters, and “street” scene in figs. S11 to S14.

### Network laser image classification

**Fig. 3.**
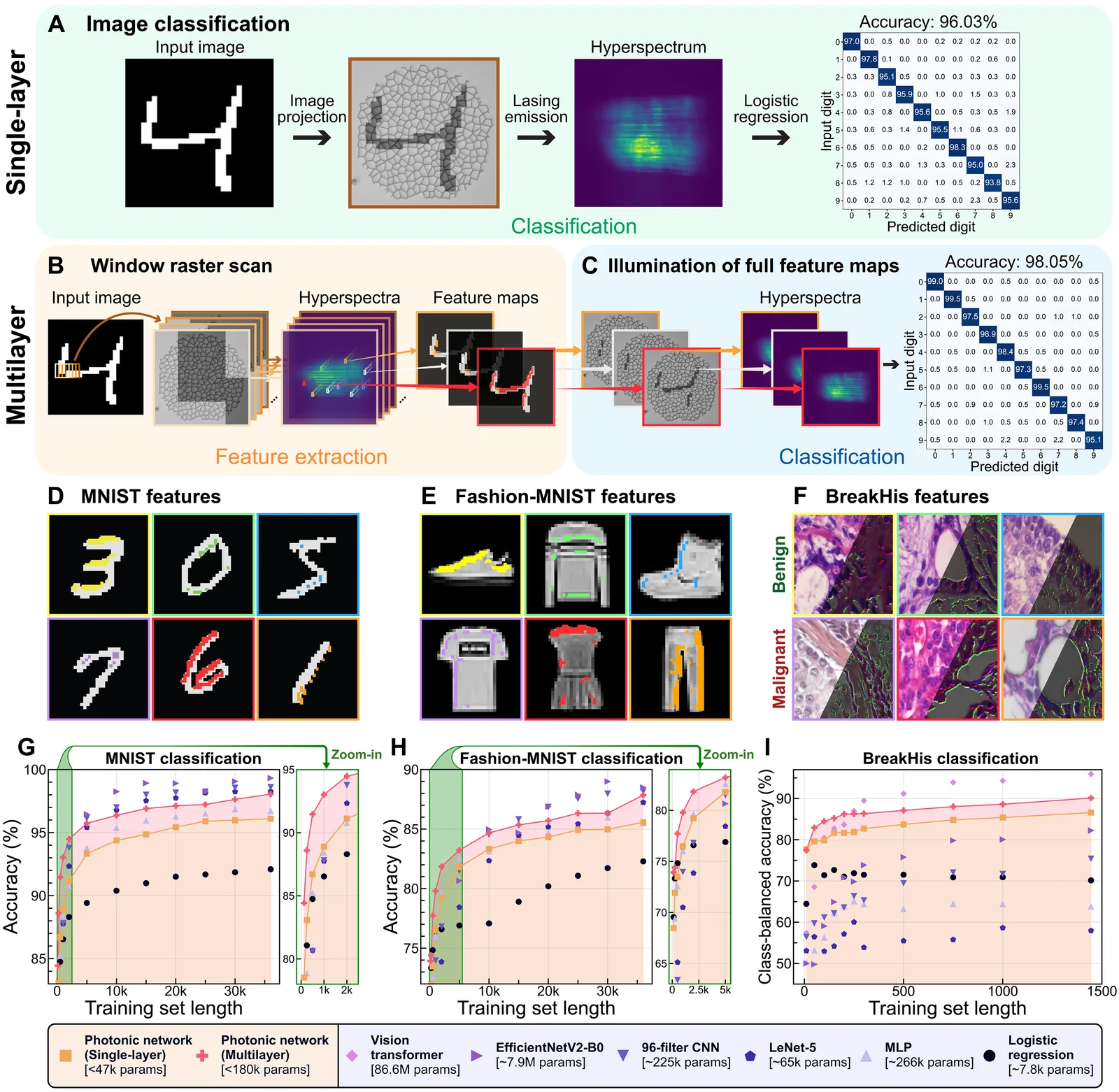
Network laser image classification. (**A**) Single-layer classification. Input images are illuminated directly onto the network, hyperspectra are measured, and a single logistic regression step is applied to the spectra to provide classification. Accuracy of 96.03% is observed for MNIST-digits. (**B** and **C**) Multilayer classification. An initial feature detection layer (B) is implemented as described in Fig. 2, generating 10 feature maps in parallel via the amplitude of 10 lasing modes (indicated by colored boxes on the hyperspectra). A second classification layer (C) is then implemented by illuminating the feature maps sequentially and measuring hyperspectra. The hyperspectra are combined, and a single logistic regression step applied to return classification predictions. Accuracy of 98.05% is observed for MNIST-digits. (**D** to **F**) Three classification tasks are implemented, with example images for each task showing detected features: (D) MNIST-digits, (E) Fashion-MNIST, and (F) BreakHis cancer diagnosis (400× magnification). (**G** to **I**) Classification accuracy versus training set length, comparing single- and multilayer photonic network accuracy against software benchmarks: logistic regression, MLP, LeNet-5 CNN, a 96-filter CNN, EfficientNetV2-B0 CNN (3040 filters, in a transfer-learning scheme with pretrained ImageNet weights), and a vision transformer (ViT) with ∼86 million parameters. Maximum accuracies for the multilayer photonic network of Digits: 98.05%, Fashion: 87.85%, and BreakHis: 90.12% are observed. Zoomed-in regions of the few-shot regime are shown in (G) and (H), where the photonic network exceeds software benchmark performance. Image licenses: MNIST-digits, CC BY 3.0 https://creativecommons.org/licenses/by/3.0/; Fashion-MNIST, MIT https://mit-license.org/https://mit-license.org/; BreakHis, CC BY 4.0 https://creativecommons.org/licenses/by/4.0/.

Here, we demonstrate the ability of the network laser to classify images. The network can be operated in a single-layer scheme, directly projecting images onto the network (Fig. 3A), or a two-layer scheme with an initial feature-detection layer (Fig. 3B), followed by projecting the feature maps onto the network (Fig. 3C). In both cases, a logistic regression step follows the final layer of hyperspectral output to generate classification predictions.

We perform classification on three tasks: MNIST-digits, Fashion-MNIST, and the challenging low-data BreakHis breast cancer diagnosis (*53*) (color histopathology images, 400× magnification, 700 × 460 resolution). Figure 3 (D to F) shows example images for each task, with colored lines illustrating feature maps detected by the network. For MNIST-digits and Fashion, we experimentally process 40,000 training images with a test set of 4000 images and training sets of 125 to 36,000 images. For BreakHis, we process 1693 images, with a test set of 250 and training sets of 10 to 1,443 images. BreakHis contains a roughly 2:1 ratio of malignant to benign images, with this class imbalance increasing task difficulty. No augmentation is performed on the original datasets to synthetically increase their length.

Single-layer classification (Fig. 3A) works by projecting whole images onto the network, measuring the hyperspectrum, then applying logistic regression on the training set hyperspectra to learn a fixed weight (per image class) for each channel of the hyperspectral output. This feed-forward single layer is similar in some respects to an Extreme Learning Machine, where input information is nonlinearly transformed and projected to a high-dimensional state by a fixed set of complex, nonlinear nodes, and then transformed to a useful computational output via a linear readout layer (*54*). However, the ability of the network laser to directly produce computationally relevant outputs such as image feature maps from the intrinsic nonlinear photonic dynamics, without requiring a trained linear readout layer, means that our system goes beyond the definition of an extreme learning machine. Once the system is trained, unseen images are classified by performing a weighted sum over the hyperspectral channel amplitudes and learned weights for each class. The class with the largest weighted sum is returned as the network prediction. The single-layer scheme obtains accuracy of 96.03% on MNIST-digits, 85.18% on Fashion-MNIST, and 86.59% on BreakHis cancer diagnosis, with measurement throughput up to 100 Hz.

Multilayer classification begins with feature detection (Fig. 3B). Ten spectral regions performing feature detection were selected, producing 10 feature maps per input image. Regions returning clear edge detection were hand selected, with future improvement available by training the feature selection process. Three feature maps are shown schematically in Fig. 3B), with all feature maps for a range of MNIST-digits and Fashion-MNIST shown in figs. S15 and S16. These feature maps are then re-illuminated on the InP network as whole images and their corresponding hyperspectra recorded, illustrated in Fig. 3C). The hyperspectral outputs from the second-layer feature map illuminations are combined in parallel, and a single logistic regression step is applied to produce classification results. The multilayer scheme obtains accuracy of 98.05% on MNIST-digits, 87.85% on Fashion-MNIST, and 90.12% on BreakHis cancer diagnosis. Further details of the regression process are given in Materials and Methods.

Network laser performance relative to software benchmarks is assessed in Fig. 3 (G to I). Classification accuracy versus training set length is assessed for single- and multilayer network laser schemes against a range of software neural networks. Five software approaches are considered for all tasks: logistic regression on the raw images (a direct control, effectively bypassing our physical system), a multilayer perceptron (300 × 100 neurons), a LeNet5 CNN (22 filters), a larger CNN (96 filters), and the much more powerful pretrained “EfficientNetV2-B0” (*51*, *52*) CNN (3040 filters, 7.9 million parameters). For the harder BreakHis 400X task with limited data, we also benchmark using a far larger and more powerful pretrained vision transformer network (*7*, *39*, *55*) (“ViT_b_16”) with ∼86 million parameters, representing a large modern software vision network. Both “EfficientNetV2-B0” and the vision transformer were trained using a transfer learning approach, initialized with pretrained ImageNet weights, which are then updated during training. Full details of the software networks are provided in Materials and Methods. We also benchmark against a “quasi-isomorphic” software network: an initial convolutional filter layer of 10× 4 × 4 filters followed by 30,000 randomly weighted ReLu neurons and a final logistic regression layer, mirroring the structure of our system. Results are shown in figs. S23 and S24 for MNIST-digits and Fashion-MNIST, respectively.

For the few-shot/low-data performance, the photonic network laser excels when training data are scarce, a regime where conventional AI can struggle. The network outperforms all considered software models in the MNIST-digits and Fashion-MNIST tasks on training sets smaller than 5000 images (digits) and 15,000 images (fashion). On the harder biomedical BreakHis task, the network outperforms the same software benchmarks, including the powerful EfficientNet CNN, across all training lengths. Only the state-of-the-art vision transformer outperforms the photonic network at longer training lengths, with the photonic network outperforming even the vision transformer up to 200 training images, a substantial result.

We score 77.7% accuracy on BreakHis from just 10 training images (five examples per class), far higher than the software models. This highlights the effective learning provided by the nonlinear photonic dynamics and mode competition of the network laser. Here, we compare performance when using only the raw training set. Software approaches can achieve higher scores, including on the BreakHis task (*56*), but this requires costly extra steps including artificial augmentation of the training set, and extensive fine-tuning of model weights, which we do not perform in our photonic scheme.

The origin of this strong few-shot performance may lie in the system’s high-dimensional, heterogeneous nonlinear responses. Recent theoretical work has shown that if a learning network is sufficiently “high dimensional” (e.g., it has a large number of output features/channels with unique nonlinear responses), then the network may learn to generalize/accurately classify from very few training examples (*11*). This behavior is exhibited in our network: A broad range of unique nonlinear responses is provided by the many lasing modes, shown experimentally in Fig. 1G). The diverse nonlinearity in the “photonic neurons” (our lasing modes) provides both high dimensionality and neuronal heterogeneity, which has been shown to promote robust learning and stronger task performance in models of biological neurons (*23*).

### Combined biomedical image segmentation and class-imbalanced diagnostic classification

**Fig. 4.**
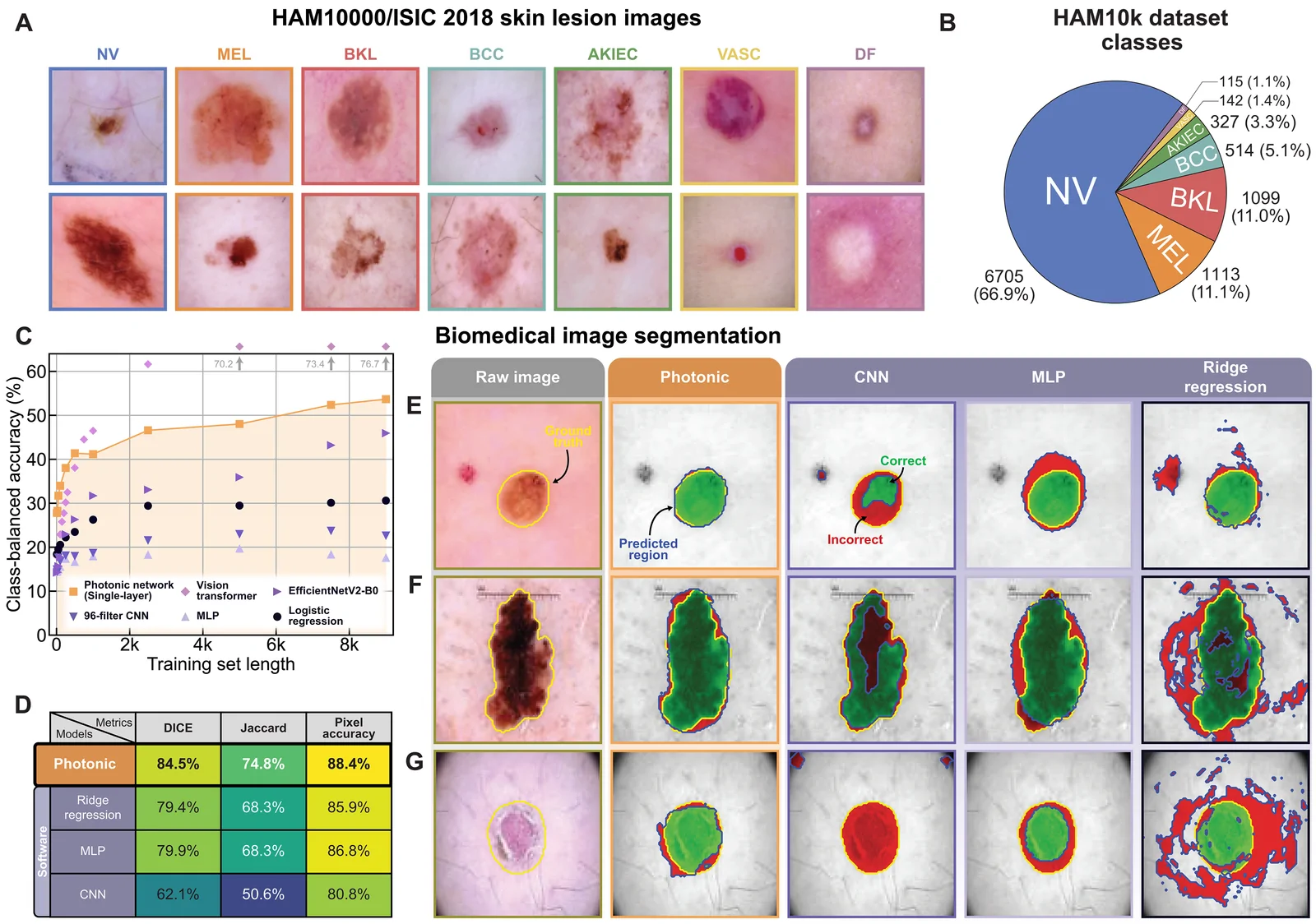
Biomedical image segmentation and class-imbalanced diagnostic classification on the HAM10k/ISIC 2018 dataset. (**A**) Example images of the seven skin lesion classes contained in the dataset. Images are complex with different instances of the same class looking very different, hard to diagnose without expert medical training. (**B**) Pie-chart illustrating the heavy class-imbalance in the dataset. (**C**) Class-balanced accuracy versus training set length, comparing photonic network against software benchmarks. Test set is fixed at 1015 images. Class-balanced accuracy penalizes incorrect classification on rarer classes more heavily, ensuring performance is not dominated by majority classes. Photonic network scores 53.7% maximum accuracy. (**D**) Segmentation scores: DICE, Jaccard, and pixel accuracy of photonic network and software benchmarks. (**E** to **G**) Image segmentation results, comparing photonic network performance against software benchmarks for three example test set images. Ground-truth masks (yellow line) generated by a human medical expert (*57*) are used for supervised training, and assessment of network performance on test images. Networks generate predicted segmentation masks (blue line) around where they think the lesion boundaries are. Green regions indicate correct segmentation where the network prediction agrees with the ground truth, and red regions show combined false positive and negative errors. HAM10k images license: CC BY 4.0, https://creativecommons.org/licenses/by/4.0/.

The neuromorphic capabilities of the photonic network are not restricted to feature detection and classification. Here, we demonstrate the ability of the network to spatially map regions of interest in biomedical images, drawing masks over skin lesions in color photographic images from the HAM10k/ISIC 2018 dataset (Fig. 4A; 10,015 images of seven different classes of skin lesion) (*57*, *58*). This task, termed segmentation, demonstrates that while the hyperspectral output of our network only contains information on the wavelength and 1D spatial position of the lasing response, it is capable of physically encoding and processing complex 2D spatial information, required for segmentation and other spatial processing. The task here is challenging: Lesions are nonuniform in shape and in a variety of colors and intensities, and many images contain darker nonlesion regions such as hairs, moles, and areas of uneven lighting. We combine two distinct tasks on the HAM10k dataset: class-imbalanced diagnostic classification, separating lesions into seven classes, and segmentation, drawing spatial masks to indicate lesion location. This twofold processing has potential future impact in automated/semiautomated combined diagnosis and surgery systems. We only need to input the HAM10k images to our photonic network once to achieve both tasks, simply retraining our computationally cheap linear output regression layer to change between diagnosis and segmentation.

#### Class-imbalanced diagnosis

Here, we diagnose lesion images into seven classes. This is made highly challenging by the heavy class imbalance of the dataset, illustrated in Fig. 4B. Machine learning typically struggles with class imbalance, tending to incorrectly classify rare classes as examples of the more common classes. Overcoming this requires costly techniques such as data augmentation, e.g., generating many distorted copies of training examples and using them to artificially extend the training set. The HAM10k task is heavily imbalanced, with the “NV” class representing 66.9% of the dataset, and four of the seven classes each representing 5.1% or less. Figure 4C shows class-balanced classification accuracy against training set length (class-balanced accuracy penalizes incorrect guesses on rare classes more heavily), with the photonic network scoring 53.7% maximum accuracy, outperforming all software benchmarks other than the vision transformer across all training lengths, and outperforming the vision transformer at training lengths up to 500 images. This demonstrates that our photonic system exhibits strong advantages on both few-shot learning and heavily imbalanced datasets, a hard problem with real-world benefits.

#### Biomedical segmentation

For segmentation, we use the same hyperspectral responses from the lesion images that we use for classification. Here, rather than using the diagnostic class as training/testing labels, and logistic regression as an output layer, we use the ground-truth segmentation maps as training/testing labels and ridge regression as an output layer (as the segmentation task has continuous output, rather than discrete labels). Otherwise, the procedure follows that used for classification. Figure 4D compares three segmentation metric scores across the test set: DICE (F1 score for segmentation), Jaccard (intersection over union), and pixel accuracy (correct pixels over incorrect). DICE and Jaccard provide the best indication of segmentation quality. Pixel accuracy scores tend to be distorted by whether the correct ground-truth mask occupies a small or large fraction of the total image. The photonic network scores highest on all metrics, highlighting the ability of the network to implement spatial processing.

Figure 4 (E to G) shows segmentation results for the photonic network and software benchmarks for three test images. In the top row (E), the photonic network accurately predicts the lesion location, ignoring the nonlesion mole, unlike the CNN and ridge regression software models. In the middle row (F), the photonic network outperforms software on a complex lesion shape with regions of varying intensity, and in the bottom row (G), the photonic network avoids incorrectly classifying the dark corner regions or hairs.

This work highlights the benefit of introducing complex heterogeneous physical nonlinearities to photonic computing systems, particularly the combination of inhibitory and excitatory dynamics and spatially distributed disordered mode profiles. By leveraging the ability of the disordered lasing network to provide heterogeneous nonlinear emission in both spatial and spectral domains alongside parallel feature extraction, we perform a range of vision tasks including feature detection, image classification, and segmentation, with promising performance relative to software approaches when learning with scarce training data. This bears interesting links with prior work exploring heterogeneous nonlinearity in spiking neural networks (*23*) and nanomagnetic systems (*59*), suggesting that engineering heterogeneous nonlinear dynamics across a broad range of photonic and nonphotonic systems may provide fertile ground for the community to explore, with potential impact on biomedical sensing, edge computing, and beyond. As the lasing modes responsible for feature detection spatially overlap, we provide parallel feature detection of multiple image features in a single network with a small on-chip footprint (with additional off-chip optical components). In contrast, existing physical convolutional hardware schemes typically detect one feature per physical repeat of the device; hence, the spatial footprint, fabrication complexity, and energy consumption typically scale with the number of features detected.

Strong learning in the few-shot/low-data regime with scarce training examples is a hard and open challenge in machine learning. The performance of our photonic network in this regime, including on hard biomedical tasks such as BreakHis and HAM10k, provides valuable evidence that physics-based neuromorphic systems can handle complex, data-constrained learning tasks. These results bring physical neuromorphic schemes closer to offering viable alternatives to conventional software-based approaches. Our good performance here extends to heavily class-imbalanced datasets, which are similarly challenging for conventional software AI. One of the key proposed applications for neuromorphic computing hardware is “edge computing,” where remotely deployed hardware performs computation directly at the point of sensing. While edge computing is often [although increasingly not solely; (*40*, *41*)] limited to inference, our system shows promise for edge deployments where both training and inference are performed, which requires adapting on-the-fly to incoming data and changing environmental conditions without access to large offline/cloud-based training data. Here, it is highly advantageous to perform well with short and imbalanced training sets, as locally collected data will typically be far from the ideal large and class-balanced training sets where software AI is most comfortable.

A key result of this work is demonstrating the importance of including both excitatory and inhibitory interactions in neuromorphic hardware. In biological systems, both of these interactions and their interplay are crucial to providing neuronal functionality (*18*, *20*, *22*), and existing physical neuromorphic hardware platforms often focus solely on excitatory signals. By engineering inhibitory nonlinear mode-competition between network lasing modes, we demonstrate the functional benefits of bioinspired inhibitory dynamics.

As we develop finer control over the network dynamics, one can envision networks engineered to detect arbitrary specific features or provide reconfigurable detection via altering network dynamics through electrical control or selective illumination of network subregions (*34*) to provide effective tunable physical weights in the input space (*60*). More broadly, our approach shows the benefits of designing physical computing systems with diverse physical nonlinearities, and a balance of competing excitatory and inhibitory interactions. While our system currently exhibits strongest performance on harder, lower training data tasks, we foresee that future iterations will extend this performance to larger datasets via training of the physical dynamics and optimization of the network graph topology.

## MATERIALS AND METHODS

Full details of the software networks are provided in Tables 1 to 6 describing software baseline network parameters and training details.

**Table 1.** CNN training details and parameters.

Parameter	Value
Total samples ($N_{\text{TOTAL}}$)	40,000
Training samples ($N_{\text{TRAIN}}$)	36,000
Test samples ($N_{\text{TEST}}$)	4,000
Validation fraction (${\text{VAL}}_{\text{FRAC}}$)	0.10
Epochs	20
Batch size	128
Learning rate	10^−3^
Optimizer	Adam
Number of trials ($N_{\text{TRIALS}}$)	10
Split seed	12345
Trial seeds	$1000+i,\;i=0,1,\ldots N_{\text{TRIALS}}$
Initialization	Xavier (PyTorch)
Training hardware	NVIDIA 4070 GPU

**Table 2.** Model description of multilayer perceptron.

Layer	Type	Neurons	Activation	Trainable parameter calculation	Trainable parameters
Input	Input	784	None	–	0
Hidden 1	Dense	300	ReLU	(784 + 1) × 300	235,500
Hidden 2	Dense	100	ReLU	(300 + 1) × 100	30,100
Output	Dense	10	Softmax	(100 + 1) × 10	1,010
**Total**					**266,610**

**Table 3.** Model description of LeNet-5 CNN.

Layer	Specifications	Activation	Trainable parameter calculation	Parameters
Input	Input image (28 × 28 × 1)	None	–	0
Conv. 1	6 filters, 5 × 5 kernel	ReLU	(5 × 5 × 1 + 1) × 6	156
Max. pool 1	Max pool 2 × 2, stride 2 × 2	None	–	0
Conv. 2	16 filters, 5 × 5 kernel	ReLU	(5 × 5 × 6 + 1) × 16	2,416
Max. pool 2	Max pool 2 × 2, stride 2 × 2	None	–	0
Flatten	Flatten layer	None	–	0
FC 1	120 neurons	ReLU	(5 × 5 × 16 + 1) × 120	48,120
FC 2	84 neurons	ReLU	(120 + 1) × 84	10,164
Output	10 neurons	Softmax	(84 + 1) × 10	850
**Total**				**61,706**

**Table 4.** Model description of larger CNN model (96 filters).

Layer	Specifications	Activation	Trainable parameter calculation	Parameters
Input	Input image (28 × 28 × 1)	None	–	0
Conv. 1	32 filters, 3 × 3 kernel	ReLU	(3 × 3 × 1 + 1) × 32	320
Max. pool 1	Max pool 2 × 2	None	–	0
Conv. 2	64 filters, 3 × 3 kernel	ReLU	(3 × 3 × 32 + 1) × 64	18,496
Max. pool 2	Max pool 2 × 2	None	–	0
Flatten	Flatten layer	None	–	0
FC 1	128 neurons	ReLU	(1600 + 1) × 128	204,928
FC 2	10 neurons	Softmax	(128 + 1) × 10	1,290
**Total**				**225,034**

**Table 5.** Description of EfficientNetV2-B0 transfer learning model.

Layer	Specifications	Activation	Trainable parameter calculation	Parameters
Input	Input image (224 × 224 × 3)	None	–	0
Stem Conv	32 filters, 3 × 3 kernel, stride 2	Swish	Included in base	–
Fused-MBConv	Stage 1: 1 block, 16 filters, expansion 1	–	Included in base	–
	Stage 2: 2 blocks, 24 filters, expansion 4			
	Stage 3: 2 blocks, 32 filters, expansion 4			
MBConv	Stage 4: 3 blocks, 48 filters, expansion 4	–	Included in base	–
	Stage 5: 5 blocks, 64 filters, expansion 6			
	Stage 6: 8 blocks, 128 filters, expansion 6			
	Stage 7: 1 block, 160 filters, expansion 6			
Head Conv	1280 filters, 1 × 1 kernel	Swish	Included in base	–
Global avg pool	Reduces spatial dimensions to 1 × 1	None	–	0
EfficientNet base	Pretrained EfficientNetV2-B0 backbone	–	Backbone total	≈ 7,200,000
FC 1	512 neurons	ReLU	(1280 + 1) × 512	655,872
FC 2	128 neurons	ReLU	(512 + 1) × 128	65,664
Output	10 neurons	Softmax	(128 + 1) × 10	1,290
**Total**				≈**7,922,826**

**Table 6.** Description of vision transformer (ViT-B/16) transfer learning model.

Layer	Specifications	Activation	Trainable parameter calculation	Parameters
Input	Input image (224 × 224)	None	–	0
Patch embedding	Patch size: 16 × 16, *D* = 768	None	Included in trunk	–
	1 class token + 196 pos. embeddings			
Transformer encoder	12 blocks, 12 attention heads	–	Included in trunk	–
	MLP hidden size: 3072			
	LayerNorm and residuals			
Transformer trunk	Vision transformer core (ViT-B/16 base)	–	86,567,656–769,000	85,798,656
Classification head	Linear layer (*K* = 2 classes)	Softmax	(768 + 1) × 2	1,538
**Total**				**85,800,194**

### Experimental methods

#### Fabrication

The InP networks are fabricated on a Si chip with a 2-μm buffer layer of SiO_2_ according to (*30*). To obtain a defect-free InP layer on SOI, we used a direct wafer bonding approach where the 127-nm-thick InP layer is grown on a sacrificial III-V semiconductor wafer, with lattice-constant matched growth. This wafer is then annealed onto the SOI wafer, and the sacrificial wafer was removed by wet etching, resulting in an InP on SOI wafer that is cleaved into chips. The network structures are then patterned into a spin-coated hydrogen silsesquioxane (HSQ) mask with electron beam lithography. The pattern is transferred into the InP layer using inductively coupled plasma (ICP) reactive ion etching (RIE) (*30*) using a cyclic two-stage process: a CH_4_ and H_2_ plasma stage for etching, followed by O_2_ plasma stage for cleaning. To reduce surface state recombination losses and oxidation, the structures are passivated with phosphoric acid and coated with 3 nm of Al_2_O_3_ using atomic layer deposition (ALD) (*61*).

#### Network design

The network was designed by using a randomly distributed point grid pattern on a plane and generating a Voronoi diagram (*29*). A Voronoi diagram is generated by forming areas around stochastically placed points that contain all points closer to a certain point than any other point. The edges of these areas form the network design. Each vertex of the designed network is formed by three waveguides. The network is cropped to a circular area with 150 μm diameter, and the density of the point patterns is varied to generate waveguide graph edges with 350 nm width and an average length of 5 μm.

#### Optical setup

The edge-detection and digit classification experiments are performed in an optical microscopy setup with a spatially structured pump (via DMD) to excite the network laser structure. A schematic of the setup is shown in the Supplementary Materials, fig. S1). The pump laser is a 633-nm femtosecond pulsed laser [Light Conversion PHAROS with ORPHEUS-VIS optical parametric amplifier (OPA) and LYRA harmonic generator, 200-fs pulse duration, 1-kHz repetition rate] and is first expanded through a telescope and shaped by reflecting on a DMD (ViALUX V-650 L). The DMD consists of 1280 × 800 programmable mirror pixels, which shape the incident uniform beam into any pixelated black-and-white image (i.e., training images). For the edge detection and MNIST classification schemes, we use the DMD only as a binary modulator, with the DMD controller thresholding the provided images at the midway point in brightness. For the projection of Fashion-MNIST and BreakHis 400X images, we use a quasi-grayscale scheme, using ordered dithering with a blue noise pattern, with multiple binary DMD pixels corresponding to one grayscale data pixel. The power of the shaped pump patterns is modulated by a motorized continuously variable neutral density (ND) filter, and the exact power received on the sample is monitored by a power meter (Thorlabs, S120VC Si photodiode, 200 to 1100 nm) on the split path reflected from a glass slide inserted. The spatially structured pump is focused on the sample through a 20× objective [Olympus Plan N 20× with a 0.4 numerical aperture (NA)]. The lasing emission from the network is collected through the same objective, filtered (Thorlabs FELH0750, 750-nm long-pass filter), and spatially and spectrally analyzed using a grating spectrometer (Princeton Instruments SpectraPro HRS-300) equipped with a 600 g/mm visible grating without an entrance slit. The signals are measured by a 2D CCD camera (Princeton Instruments Pixis 256) with a spatial resolution of ∼1 μm.

Throughput of images was run up to 100 Hz, with the current limiting factor being the camera detection. We have run experimental tests on a similar system up to 500 Hz. With the correct camera, throughputs of 1 kHz are readily feasible without modification to the scheme. Beyond this, the next limiting factor is the rate at which the DMD can display new images. Our current DMD has 10-kHz speed, and faster DMDs are available up to the megahertz, with ongoing improvements in DMD technology likely to improve this in the near future. The repetition rate of our pulsed laser goes to 100 kHz, with megahertz pulsed lasers or on-chip light sources available. The eventual fundamental limit is the 10 to 100 ps timescale of the photonic dynamics. Beyond this, more fundamental changes to the physical system/excitation scheme will be required.

A second 80-ps pulse pump laser was used to confirm that we are not limited to operating with femtosecond pulses and can operate with a range of pump wavelengths. We used the Picophotonics CP32 532-nm MOPA pump laser, which successfully pumps our InP network to lase. Experimental data confirming this (hyperspectra, LL plots) are shown in fig. S3.

#### Hyperspectral measurements

The raw hyperspectral data recorded by the CCD has 1024 spectral channels and 256 spatial channels. For feature detection, hyperspectral regions comprising four adjacent spectral channels and eight adjacent spatial channels are used to generate each feature map, with the averaged intensity taken within the hyperspectral region.

For the image classification layer, the hyperspectra are divided into averaged regions of 16 adjacent spectral channels and four adjacent spatial channels, with each region passed to the logistic regression layer and assigned a single fixed linear weight during training. The feature maps of the “Cameraman,” “CL,” MNIST, Fashion-MNIST, and BreakHis images (and “street” image in the Supplementary Materials) are generated from raster-scanning the binarized images across the kernels with a size of 4 × 4, and a stride of 1. For the ease of the data collection process and minimizing redundant data collections, a set of hyperspectral data from all possible 4 × 4 image slices (a total of 65,536 excitation patterns) is collected sequentially. The feature maps of these images are then generated by picking the corresponding hyperspectra based on the slice windows. The same process applies when we generate the edge maps in the two-layer classification scheme.

The feature maps of the mandrill image (Fig. 2K) with the kernel sizes of 4 × 4, 7 × 7, and 11 × 11 are measured separately for a consistent comparison. The feature maps with RGB channels are generated by raster-scanning every image slice from the 256 × 246 pixel image (with the size of the kernel and stride of 2) on the network laser, comprising a total of ∼45,000 sequential acquisitions for each of the kernels. The region selection and averaging performed here could be implemented by using a lower-resolution spectrometer, with the additional benefits of faster data acquisition and lower cost.

#### NetSALT simulations

The lasing modes and intensities of the network are modeled using netSALT, (*34*), which extends SALT (*48*) for quantum graphs. The netSALT model was previously developed by several of the co-authors, and a detailed derivation is provided in the supplementary information of (*34*). SALT solves the following partial differential equation, derived from the Maxwell Bloch equations that describe lasing two-level atoms interacting with light, to find the lasing mode profiles $\Psi_{\mu }\left(\vec{r}\right)$ and their frequencies $k_{\mu }$

$$\left\{\nabla^{2}+\left[\epsilon_{c}\left(\vec{r}\right)+\frac{\gamma_{a}D\left(\vec{r}\right)}{k_{\mu }-k_{a}+i\gamma_{a}})\right]\right\}\Psi_{\mu }\left(\vec{r}\right)=0$$ (1)

$$D\left(\vec{r}\right)=D_{0}\left(\vec{r}\right){\left[1+\sum_{\nu }\Gamma_{\nu }{\left|\Psi_{\nu }\left(\vec{r}\right)\right|}^{2}\right]}^{-1}$$ (2)

where $\epsilon_{c}$ is the permittivity of the medium, $k_{a}$ is the central frequency of the Lorentzian gain profile, $\gamma_{⊥}$ is the gain width, $\Gamma_{\nu }=\gamma_{a}^{2}/\left[\gamma_{a}^{2}+{\left(k_{\nu }-k_{a}\right)}^{2}\right]$ is the Lorentzian gain at the mode frequency $k_{\nu }$, $D_{0}$ is the spatially varying pump intensity, and *D* is the population inversion that is reduced from $D_{0}$ due to mode competition. NetSALT computes the lasing from networks by solving the 1D SALT equation on the edges of the network and applying continuity of fields and flux at the nodes of the network (*34*).

The designed network is represented as a graph, with the edges subdivided to overlap with an 8 × 8 grid to correspond to the kernel. The edges of the network have a complex permittivity $\epsilon_{c}={\left(3.4+0.001i\right)}^{2}$ when unpumped and a Lorentzian gain profile centered at normalized frequency $k_{a}=7$ with a width $\gamma_{a}=0.35$, and are pumped up to a normalized power $D_{0}=0.5$ [for a detailed physical explanation of the parameters, see (*34*)]. A separate simulation is performed for each 8 × 8 binary kernel, by pumping only the edges falling under the illuminated pixels. The passive (without pumping) modes of the network are first identified in the complex frequency plane *k*, and these are the same for all kernels. For each kernel, the modes are tracked in the complex plane as the pump power is increased, allowing comparison between the same modes across different kernels. As a mode reaches the real frequency plane $\left[\text{Im}\left(k_{\mu }\right)=0\right]$, it attains threshold and starts lasing. Beyond this pump power, the frequency and shape of the mode do not change, but mode competition between the lasing modes modifies their lasing intensities. They further deplete the gain available to the remaining modes, increasing their thresholds. The mode frequencies, thresholds, and intensities found by netSALT incorporate the mode competition, gain depletion, and nonlinearity.

As netSALT provides single-mode frequencies and intensities but not the widths, the realistic spectra in Fig. 2C are created by assigning a width of 0.005 to each mode and adding a broad photoluminescence background. The mode feature maps in Fig. 2F are made by selecting specific modes and plotting their intensities for the kernels corresponding to the kernel scan of Fig. 2E.

### Regression process for the network laser image classification task

Hyperspectra are recorded for the 10 feature maps for each of the 40,000 input images, with initial hyperspectral resolution of 1024 spectral channels and 256 spatial channels. The top and bottom 25 spatial channels are discarded as they fall outside the InP network, leaving 206 spatial channels. These channels are then binned using simple averaging with binning factors of 16 and 2 for the spectral and spatial channels to give 64 and 103 spectral and spatial channels, respectively, per feature map.

The spectral and spatial channels of all feature maps are then combined in parallel to produce a single 2D array, with each row of the array corresponding to a single input image and each column corresponding to a single “pixel” of the binned hyperspectra. The array is separated into separate “train” and “test” sections, with final accuracy results of 98.05% (MNIST-digits) and 87.85% (Fashion-MNIST) reported from train and test lengths of 36,000 and 4000 images, respectively.

Feature selection is performed on the data using the SelectKBest function from Sklearn, using chi-square correlation between the hyperspectral data and the ground-truth image class labels in the training set. A parameter search was performed to identify 36,000 as the number of features to keep from the initial pool of 58,000 features. After feature selection, logistic regression is performed on the training data using Sklearn’s logisticRegression function. The lbfgs solver was used with a regularization parameter of *C* = 6.8, identified using a parameter search.

### Regression process for the network laser image segmentation task

The process is nominally identical to the image classification workflow, save for these details on replacing logistic regression with ridge regression, and changing the ground truth from classification labels to segmentation maps: Ridge regression is used instead of logistic regression (using Sklearn’s built in ridge regression function) as we want continuous outputs here. Logistic regression returns discrete outputs, such as class-based classification predictions. Segmentation is performed by generating 2D maps of confidence/probability that a pixel is part of a lesion; hence, ridge regression is used as the output layer. The confidence maps are then thresholded at 50% confidence to produce binary maps, and regions with higher than 50% are predicted to contain lesions. We experimented with varying thresholds and having this as an optimized hyperparameter, but the best performing threshold values were so close to 50% (plus or minus 1% typically) that we leave the threshold fixed at 50% to simplify the process.

The ground truth labels used for training and testing in the segmentation tasks are 2D maps produced by a human medical expert (*57*). We train fixed weights on each hyperspectral output channel, which generates segmentation maps based on network hyperspectra, trained by maximizing DICE accuracy against the ground-truth maps. We train on 9000 images and test on 1015.

### Software machine learning comparisons for image classification

We benchmark our system against five software approaches for the classification tasks: logistic regression, a multilayer perceptron, a small CNN (LeNet-5), a larger CNN model with more filters (96) and trainable parameters, and a far larger EfficientNetV2-B0 CNN (7.9 million parameters, 3040 filters) pretrained using ImageNet weights and operated in a transfer learning scheme. Within the training sets, we set aside a fixed 10% validation split to monitor generalization and detect overfitting. For CNN models, weights are initialized using Xavier/Glorot initialization (biases set to zero). We train using the Adam optimizer with learning rate 10^−3^ and batch size 128, for 20 epochs, using cross-entropy loss. To ensure reproducibility, we report the full list of trial seeds and use a fixed dataset subsampling/splitting seed; results are aggregated across 10 independent trials. Figure S25 shows validation versus test accuracy on MNIST-digits for the “LeNet5” and “96-Filter CNN” software CNN baselines over training epochs, with the models converging well with stable plateaus observed and no overfitting.

All software CNN baseline training was performed on an NVIDIA GeForce RTX 4070 GPU. To address overfitting, validation accuracy versus test accuracy as a function of epoch (mean ± standard deviation across trials) for each dataset/model pair (MNIST–LeNet-5, MNIST–96-filter CNN, Fashion-MNIST–LeNet-5, and Fashion-MNIST–96-filter CNN) is monitored. The plots of validation/test accuracy over training epochs are presented in fig. S25, showing that validation and test accuracies track well during training, reaching a plateau without diverging. Overfitting issues are not observed.

#### CNN training parameters

The model descriptions and number of trainable parameters for each model are given below, and models were coded in Python using the Sklearn, keras, and PyTorch packages.

#### Multilayer perceptron

The multilayer perceptron comprises an input layer of 784 neurons (one per 28 × 28 image pixel), an initial hidden layer of 300 neurons with ReLU nonlinear activation, a second hidden layer of 100 neurons with ReLU nonlinear activation, and then a final softmax layer.

#### LeNet-5 CNN structure

The LeNet-5 CNN architecture comprises seven layers: two convolutional layers each followed by a max pooling layer, two fully connected layers, then a final softmax output layer. In total, 22 convolutional filters were used.

#### Larger CNN model (96 filters)

The larger CNN model has a more complex structure than the LeNet-5 CNN, with more kernel filters and trainable parameters. In total, 96 convolutional filters were used.

#### EfficientNetV2-B0 transfer learning model

This model uses transfer learning using the EfficientNetV2-B0 architecture with an added classification head MLP. EfficientNetV2 is a relatively modern [2019 (*51*) original EfficientNet model; 2021 (*52*) for the EfficientNetV2 used here] large CNN, frequently deployed in industrial and commercial learning applications and applied to real-world problems. EfficientNetV2-B0 has 3040 convolutional filters, far more powerful than the other CNNs considered here. As is common in learning applications without sufficiently large training sets to fully optimize network parameters from scratch, pretrained weights learned from training on the ImageNet dataset are used to initialize the network and set up a broad range of feature extracting kernels before the network is optimized on the training set of an arbitrary desired task.

The EfficientNetV2-B0 base model is followed by a 512 × 128 neuron MLP classification head to aid repurposing of the initialized ImageNet weights to arbitrary tasks, comprising global average pooling, two fully connected (dense) layers with ReLU activations, and a final softmax classification layer.

#### Vision transformer (ViT-B/16) transfer learning model

We additionally benchmark using a vision transformer (ViT-B/16) image classifier, pretrained with ImageNet weights in a transfer learning scheme (see docs.pytorch.org/vision/main/models/generated/torchvision.models.vit_b_16.html for full details). Vision transformers process an image by splitting it into fixed-size patches (here,16 × 16 on a 224 × 224 pixel image), linearly embedding each patch, adding a learnable class token and positional embeddings, and then applying a stack of Transformer encoder blocks with multihead self-attention and MLP-style linear weight/nonlinear neuron sublayers. The final class-token representation is mapped to class logits via a linear classification head (*39*).

In the transfer-learning scheme used here, images are resized to 224 × 224 and ImageNet-normalized. Training is performed with an initial head-only warm-up (backbone frozen) followed by end-to-end fine-tuning of all model weights, including the backbone and final MLP classifier head.

## Supplementary Materials

### This PDF file includes:

Figs. S1 to S25 Supplementary Text References
