# Supplementary material for: Few-shot neuromorphic vision in a nonlinear photonic network laser
Source: Sci Adv. 2026 Sep 4;12(36):eaec6546. doi: 10.1126/sciadv.aec6546 (PMC13544200; doi:10.1126/sciadv.aec6546)
Supplement: Supplementary file 1 [file sciadv.aec6546_sm.pdf]

Science Advances
AAAS

Supplementary Materials for

Few-shot neuromorphic vision in a nonlinear photonic network laser

Wai Kit Ng *et al.*

Corresponding author: Riccardo Sapienza, r.sapienza@imperial.ac.uk;
Jack C. Gartside, j.carter-gartside13@imperial.ac.uk

Sci. Adv. **12**, eaec6546 (2026)
DOI: 10.1126/sciadv.aec6546

This PDF file includes:

Figs. S1 to S25
Supplementary Text
References

Optical setup schematic

Figure S1 shows a visual schematic of the experimental setup and optical path.

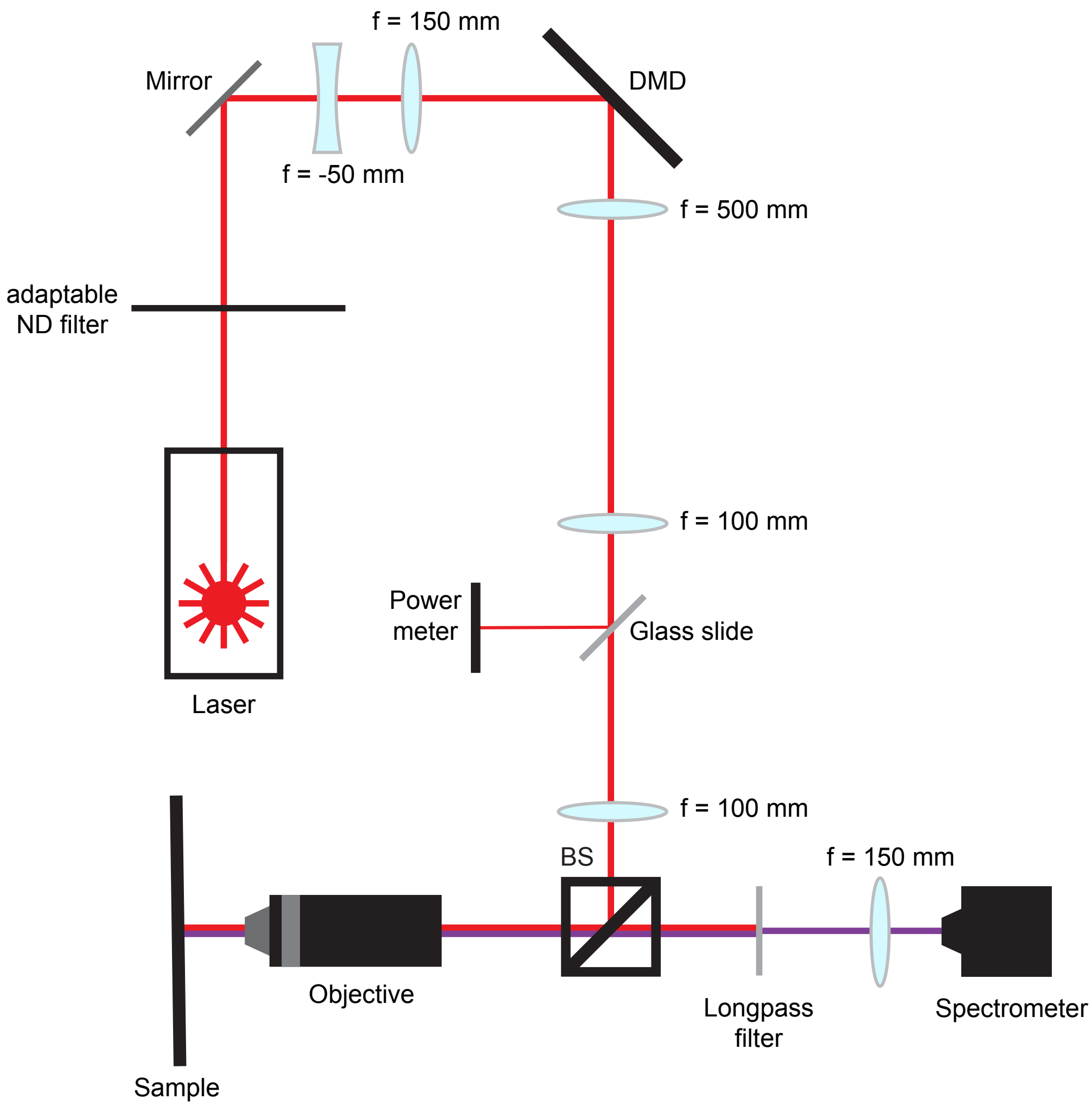

Supplementary Figure S1. Experimental optical setup. The samples are pumped using a femtosecond-pulsed laser (red path) with the power adjusted via an adaptable neutral-density (ND) filter, expanded through telescope (blue lenses), reflected off a digital micromirror device (DMD) to shape the beam pattern, and focused through a telescope and objective. A glass slide reflects a fraction of the light to a power meter for in-situ power measurement. A beam splitter (BS) enables the pump (red) and emission (purple) paths to both go through the objective. The pump is filtered out by a longpass filter and the emission is focused into a spectrometer.

Network laser image and spectral location of feature-detecting modes

Figure S2 shows details of the InP network laser structure, spatial localisation of lasing modes and the hyperpectral regions used for feature detection. Figure S2A shows an optical microscope image of the network used in this study. Figures S2 B and C show the spatial profile of the light emitted by the network in response to a spatially-structured pump illumination (dark regions of the network receive no pump illumination). Figure S2 B shows all light emitted by the networks, Fig. S2 C shows the spatial localisation of different wavelength lasing modes, with the colour-coding corresponding to the highlighted regions on the hyperspectra shown in Fig. S2 D. The colour corresponds to the scheme used for the mode feature maps throughout this work, eg. the red feature maps are formed via the amplitude of the hyperspectral region highlighted in red (labelled '5'). Figure S2 E shows the spectral position of the feature-detecting mode formed by averaging over all spatial channels.

Experimental demonstration of network lasing using picosecond pulse pump laser

To confirm that we are not restricted to pumping our InP network laser using femtosecond or 633 nm pulses, here we demonstrate experimental pumping of our network using a 532 nm 80-picosecond pulse laser, supplied by Picophotonics (model CP32). Figure S3 shows spatially-integrated lasing spectra, LL curve, and network hyperspectrum all confirming that the network lases in response to an 80-picosecond pump. The Picophotonics CP32 pump laser consumes 10.5 W, with a rep rate of 10 kHz.

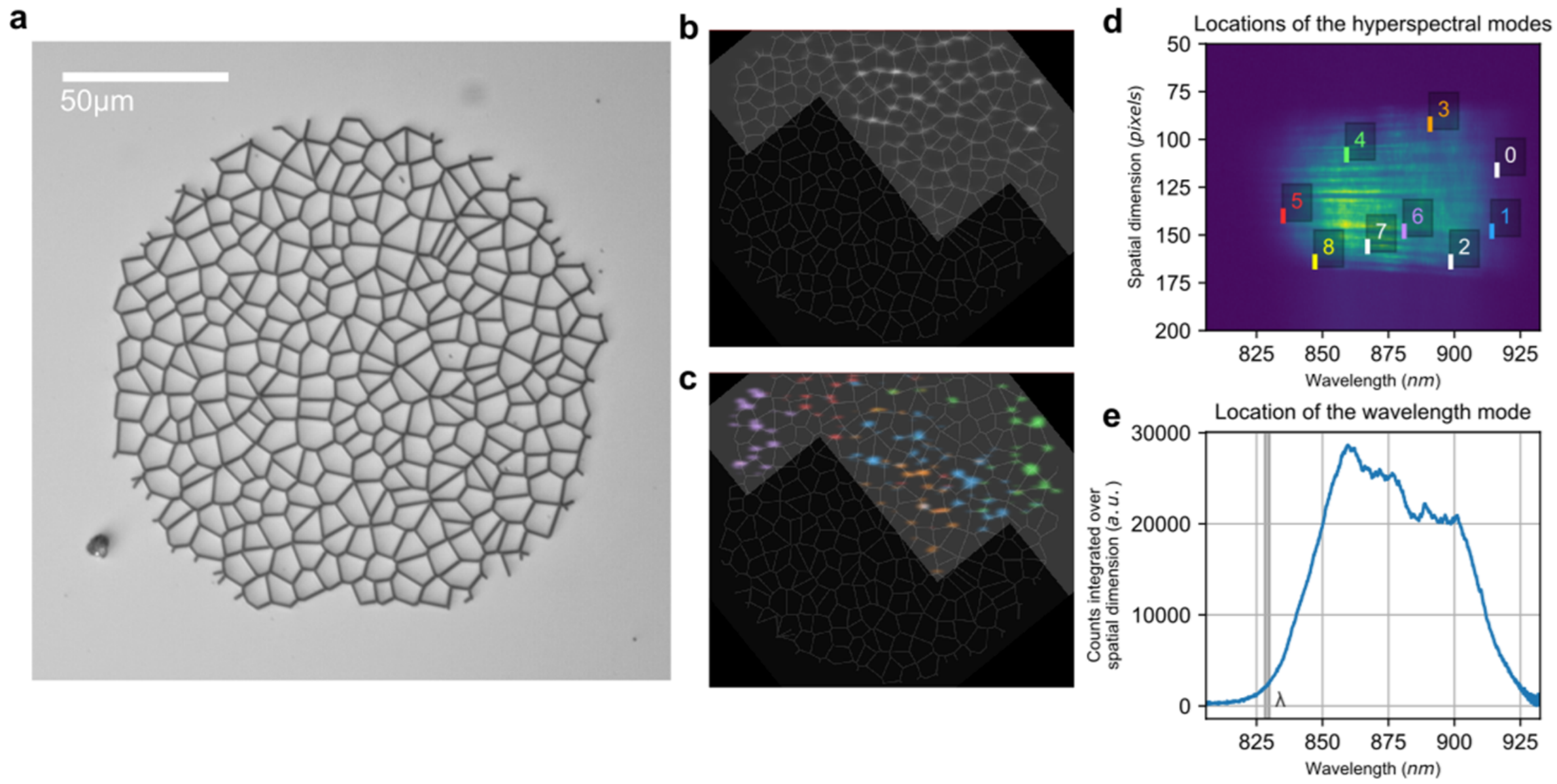

Supplementary Figure S2. Network laser image and spectral location of feature-detecting modes (A) Optical microscope image of the InP network laser used in this study.

(B) Optical image of light emitted by network under diagonal-edge illumination (dark regions of the network receive no pump illumination.

(C) Optical image from B with emitted light by the network colour-coded corresponding to the wavelength of the emitted light, with the colours corresponding to the labelled hyperspectral regions in D. Different regions of the network are seen to emit light at different wavelengths.

(D) Network hyperspectral output recorded for the pump illumination pattern shown in A,B. Nine hyperspectral regions are labelled, colour-coded in correspondence with the detected feature maps used throughout this study indicating the wavelength and spatial position of the spectral regions used for feature detection. Notably, feature detecting regions are largely positioned around the edges of the main central region of hyperspectral intensity.

(E) Position of the tenth feature-detecting spectral region, which is formed by averaging over all spatial channels and taking the amplitude within the indicated grey region.

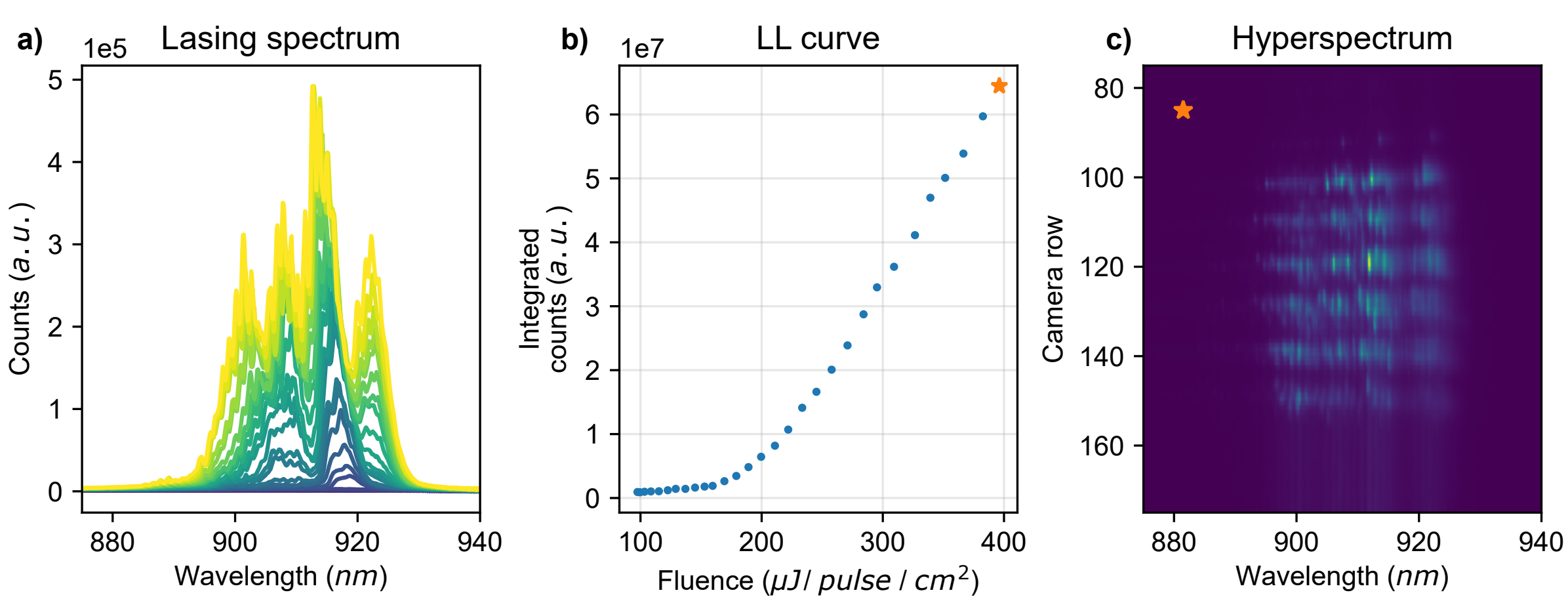

Supplementary Figure S3. Experimental demonstration of InP network lasing when pumped by an 80-ps, 532-nm pump laser

(A) Network lasing spectra (integrated spatially over whole network) in response to 80 ps, 532 nm pump (picophotonics brand laser) pulses of increasing intensity.

(B) LL curve showing onset of lasing.

(C Network hyperspectrum taken at pump fluence of 400 microjoules per pulse per cm^2 (indicated by orange star in **(B)**). Many network lasing modes are observed

NetSALT simulated feature detection - additional feature maps and spatial mode profiles

Figure S4 shows an expanded set of 28 netSALT simulated modes, spatial mode profiles and corresponding feature maps for the CL test image. A broad range of detected image features are observed.

Figure S5 shows the full set of all 172 netSALT simulated mode feature maps. Many detected features are observed, alongside modes which only respond to very specific image regions - often lasing for only a handful of pixels of the test image eg modes 62, 157 and 109. Some modes return an output close to the input image, sometimes with enhanced lasing amplitude along a specific image feature, eg modes 2, 6, 9 and 35.

Figure S6 shows the maximum intensities attained by the 172 simulated modes to illustrate their contribution to the output spectrum. Most of the modes show at least 1-10% of the maximum output intensity, with the ones highlighted in Figure S4 in the 10-100% range.

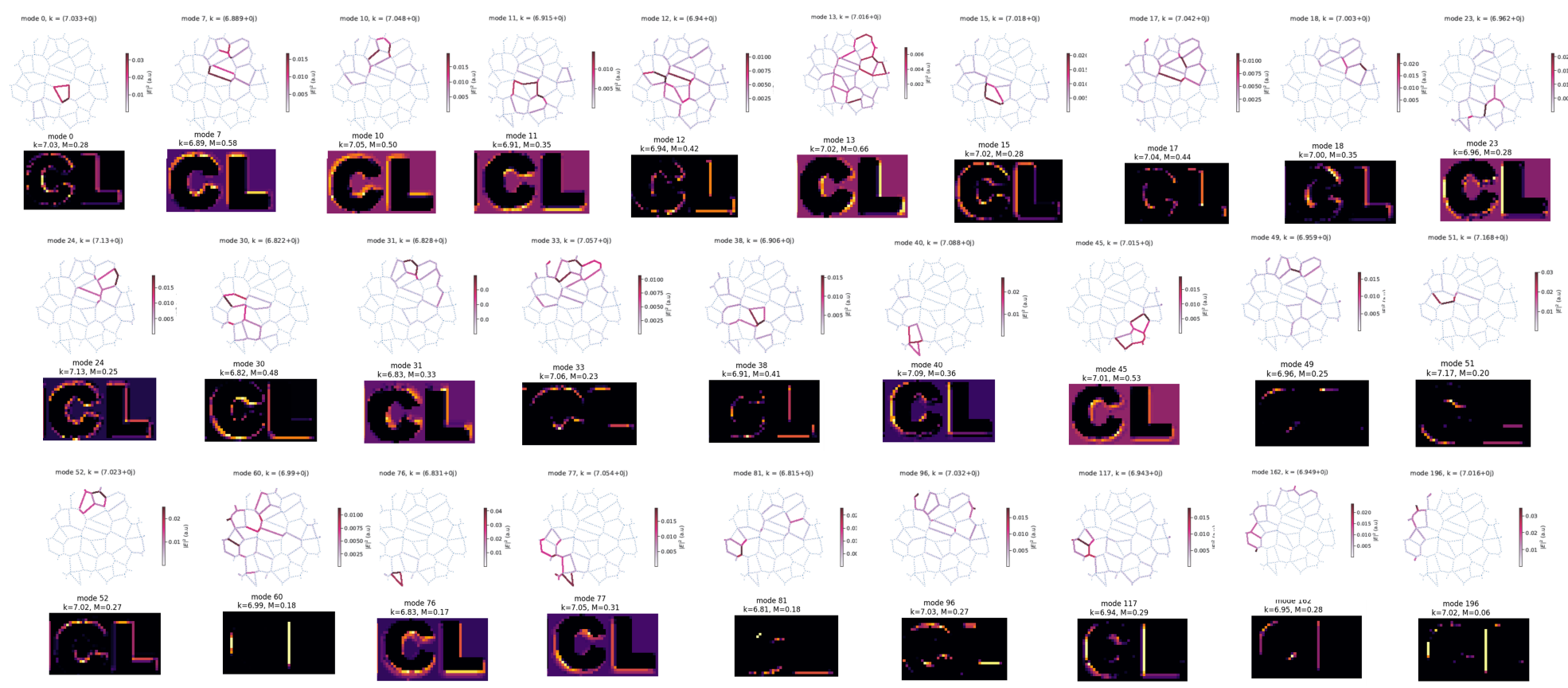

Supplementary Figure S4. Spectrally-multiplexed feature-maps and corresponding spatial mode profiles simulated via netSALT. This is an expanded set for 28 netSALT simulated modes.

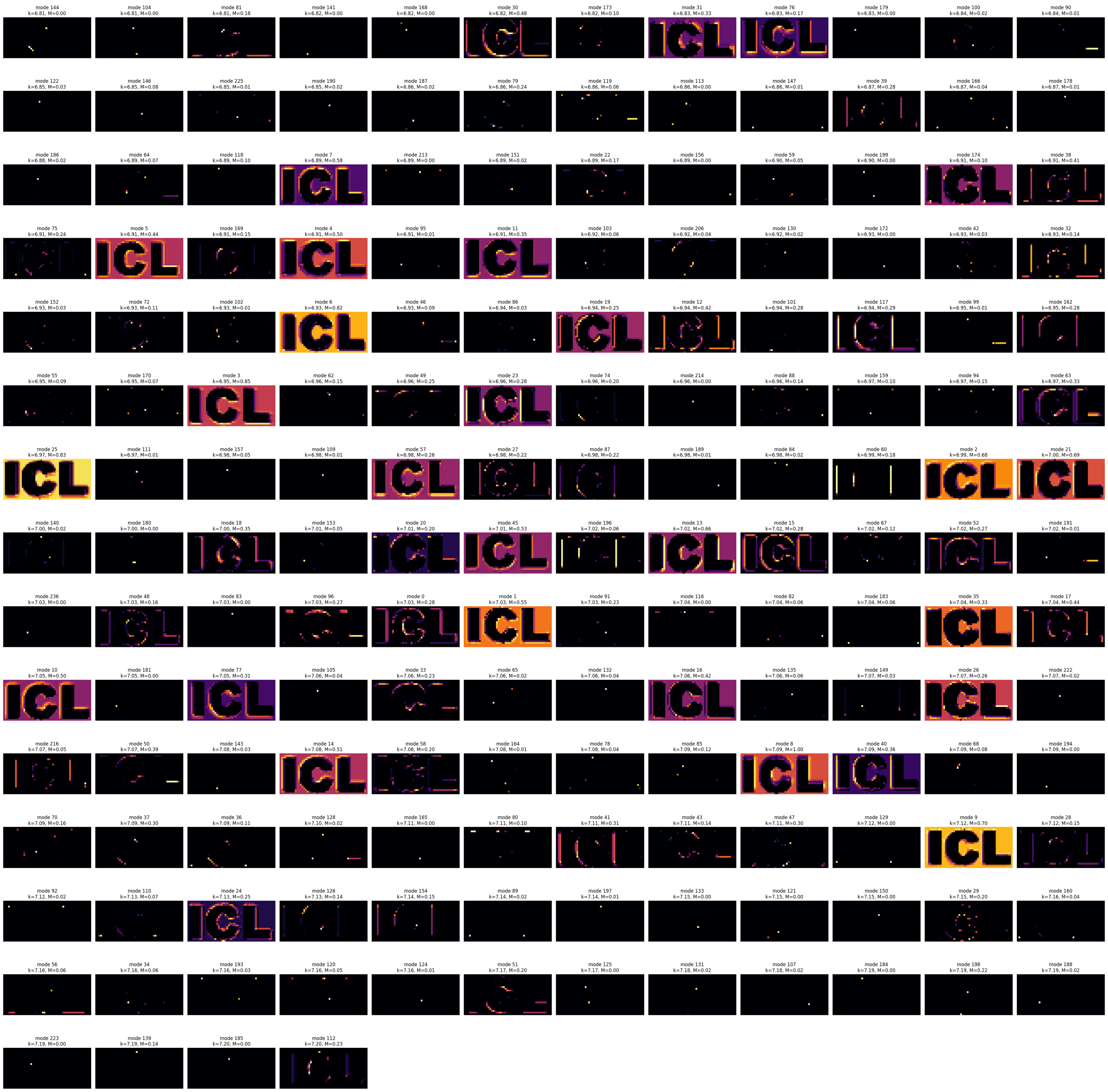

Supplementary Figure S5. Complete simulated feature maps from all 172 netSALT simulated modes.

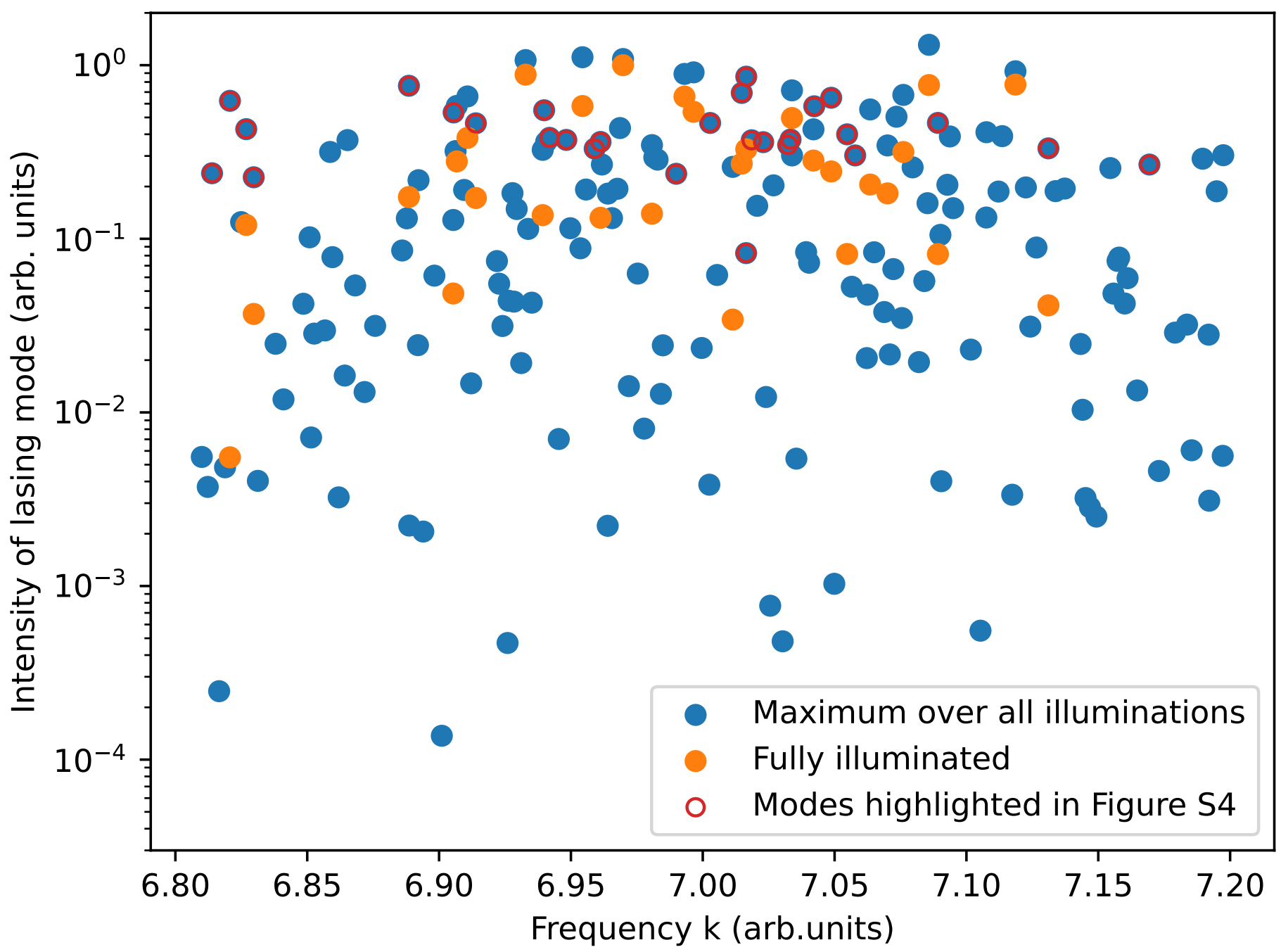

Supplementary Figure S6. Maximum intensity for the 172 netSALT simulated modes. Modes with feature maps shown in Fig. S4 are highlighted.

NetSALT simulated feature detection for a regular hexagonal network

Figure S7 shows netSALT simulated lasing spectra and feature detection for a network with a regular hexagonal graph topology. The regular network shows some ability to detect features, seen in Fig. S7C,D where the system can produce different outputs between full illumination, left/right edge illumination and top/bottom edge illumination - but the regular network can not discern the difference between left and right edges or top and bottom edges. Looking at the full range of detected feature maps in S7D, a much smaller range of features are detected relative to the irregular network.

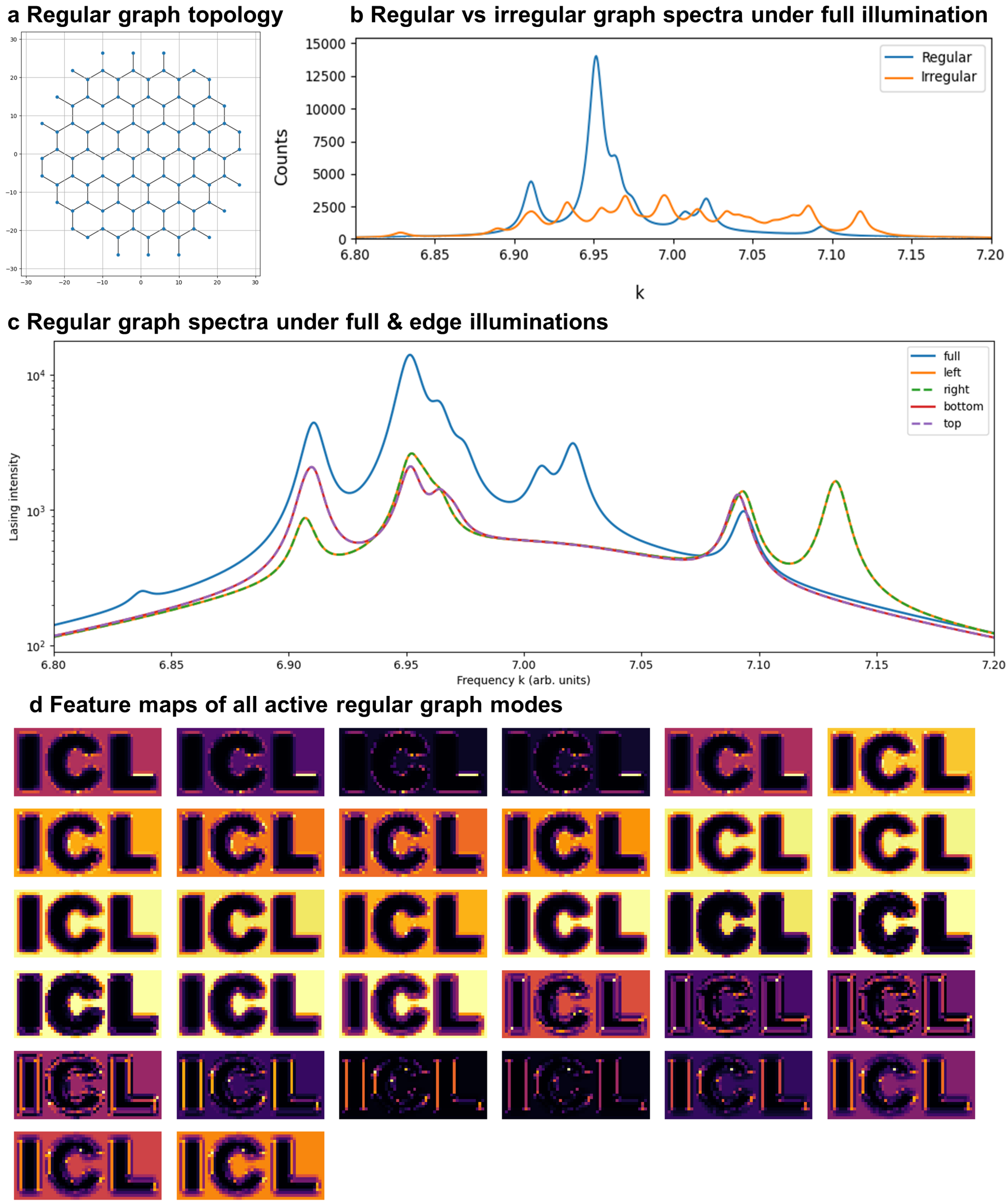

Supplementary Figure S7. netSALT simulations of a regular hexagonal network. **(A)** Regular hexagonal graph topology, 50-µm diameter graph. **(B)** Comparison of network spectra under full illumination between the regular hexagonal network and the irregular network examined in Fig. 2. **(C)** Comparison of regular hexagonal network lasing spectra under a variety of illumination patterns, full illumination and left, right, bottom and top edge illumination. The spectral response can differentiate between full illumination, left/right edge illumination and top/bottom edge illumination - but not between left and right edges, or top and bottom edges. The irregular network provides more sensitivity across a greater range of image features than the regular network. **(D)** Mode feature maps for all the active regular graph modes. Far fewer features are detected than the irregular graph.

Experimental feature detection - additional feature maps and test images

Figures S8, S9 and S10 show experimentally measured feature maps for 10 lasing modes for a range of test images to further examine the feature detection functionality.

Figures S8 and S9 show feature maps for modes 0-4 and 5-9 respectively in response to four test images: positive and negative tone circles and stars. The feature maps for these test images give a strong visual representation of the range of image features which each mode is sensitive to. Each mode responds to a distribution of similar edges, e.g. some finite arc on the circle or a range of adjacent spikes on the star. This demonstrates that the modes don't only respond to one very specific illumination pattern, but rather a distribution of similar features. This is exactly the behaviour desired for feature detection functionality, eg mode 3 responds strongly across a range of 'left' image edges, which is far more useful than only responding to a perfect vertical left edge.

Figure S10 shows the response of modes 0-9 for a range of image edges (input images in left-most column) which are gradually rotated. This provides a good visual indication of the range of image features detected by each mode, and also shows that the ten modes provide feature detection response across all angles of image edge between them.

It is important to note that while in netSALT we can spectrally isolate the lasing response of specific modes, even if multiple modes may occupy overlapping spectral regions - this is not possible in experiment. As a result, the selected experimental hyperspectral regions and corresponding feature maps may be contributed to by more than one lasing mode.

Figures S11, S12, S14, S15 and S16 show feature maps for all ten modes across the cameraman, CL and street test images, and selected MNIST-digits and Fashion-MNIST images respectively.

Figures S17, S18, and S19 show the individual mode maps used to construct the composite RGB maps in Fig. 2k).

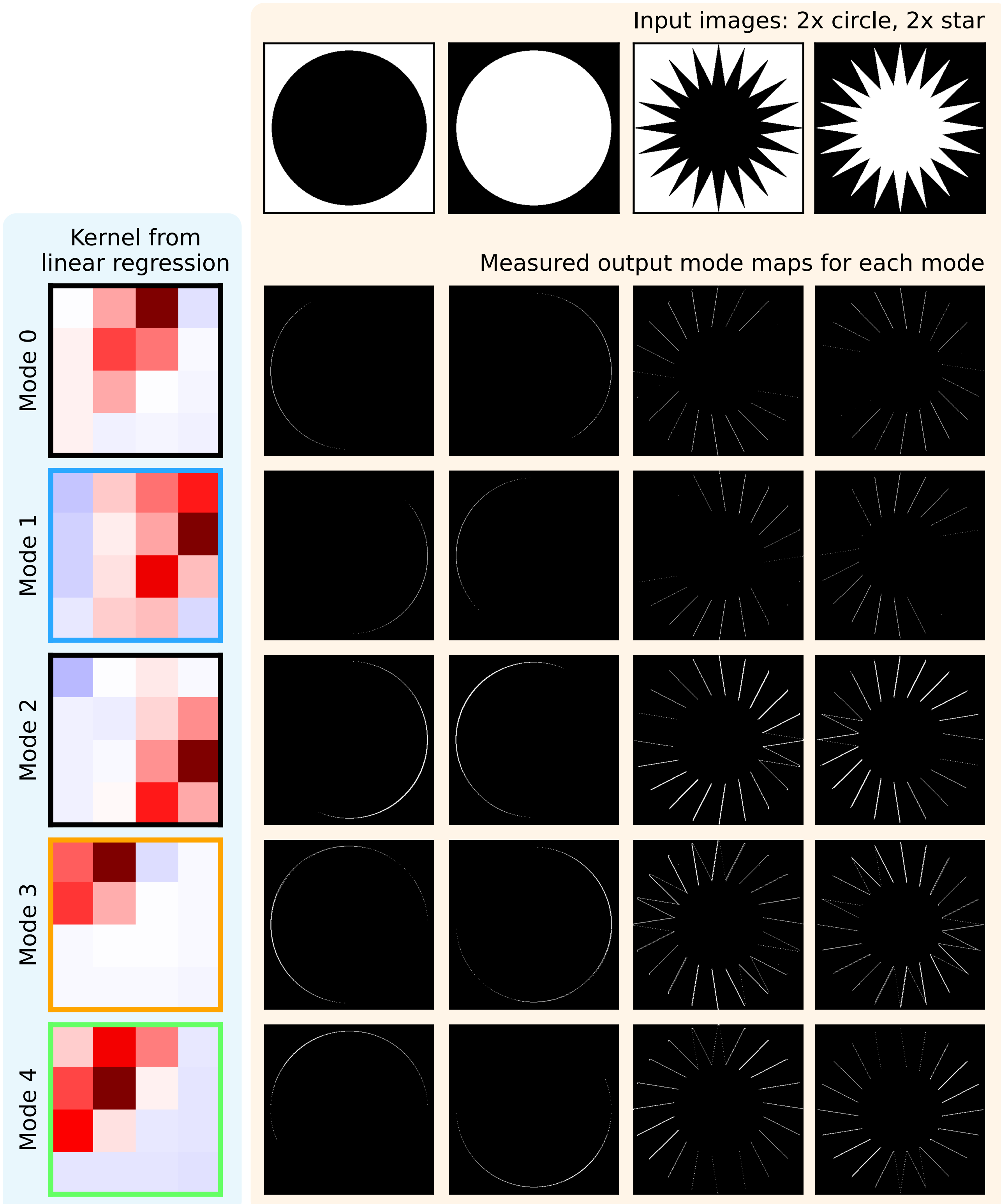

Supplementary Figure S8. Experimental feature maps and learned equivalent kernels for modes 0-5 on four test images, of black and white circles and stars. Each mode has a kernel displayed which was learned by comparing input images to experimentally measured feature maps, and learning the effective kernel via linear regression.

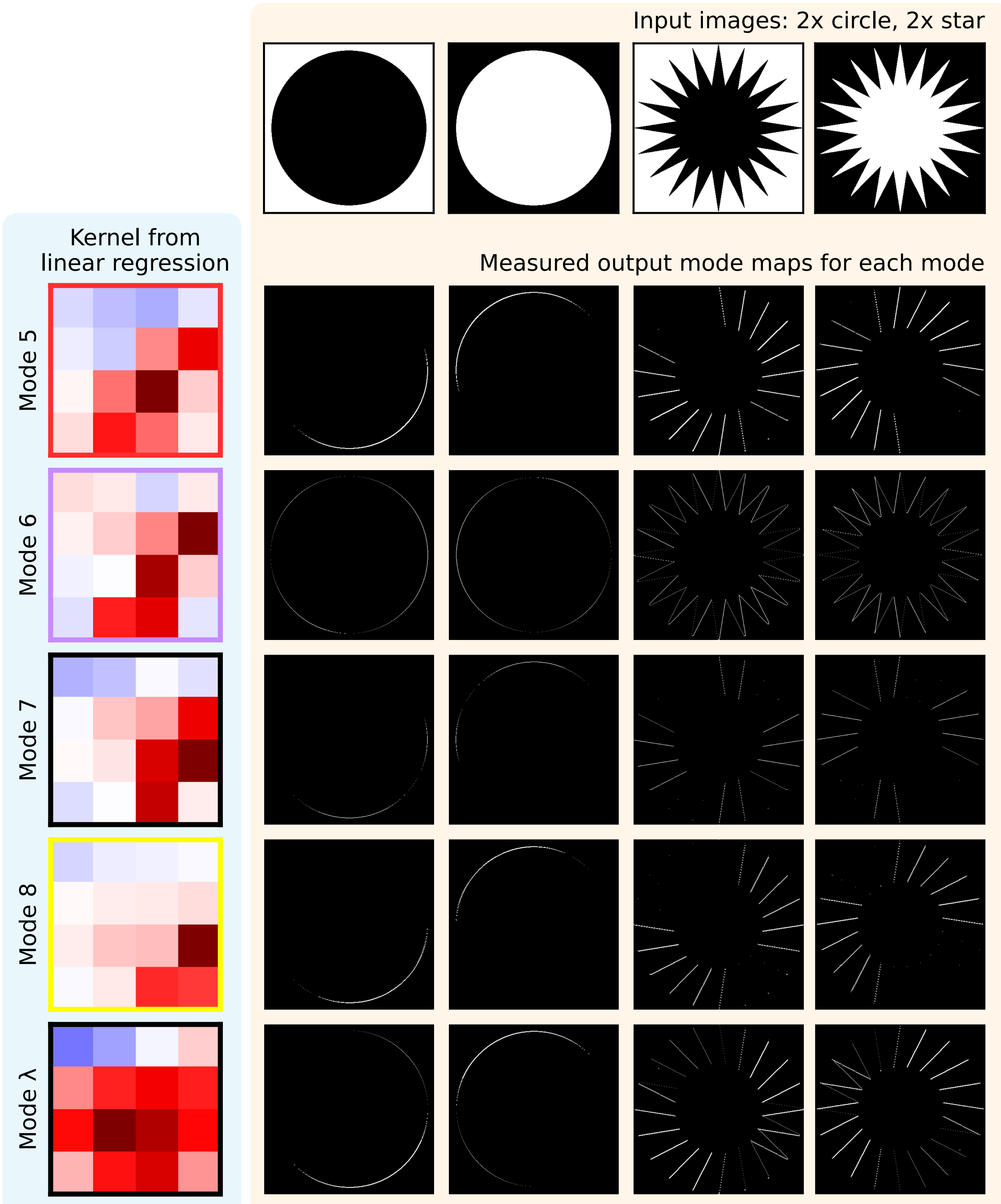

Supplementary Figure S9. Experimental feature maps and learned equivalent kernels for modes 6-9 on four test images, of black and white circles and stars (mode 9 integrates over all spatial channels, hence is labelled lambda as it contains no spatial information). Each mode has a kernel displayed which was learned by comparing input images to experimentally measured feature maps, and learning the effective kernel via linear regression.

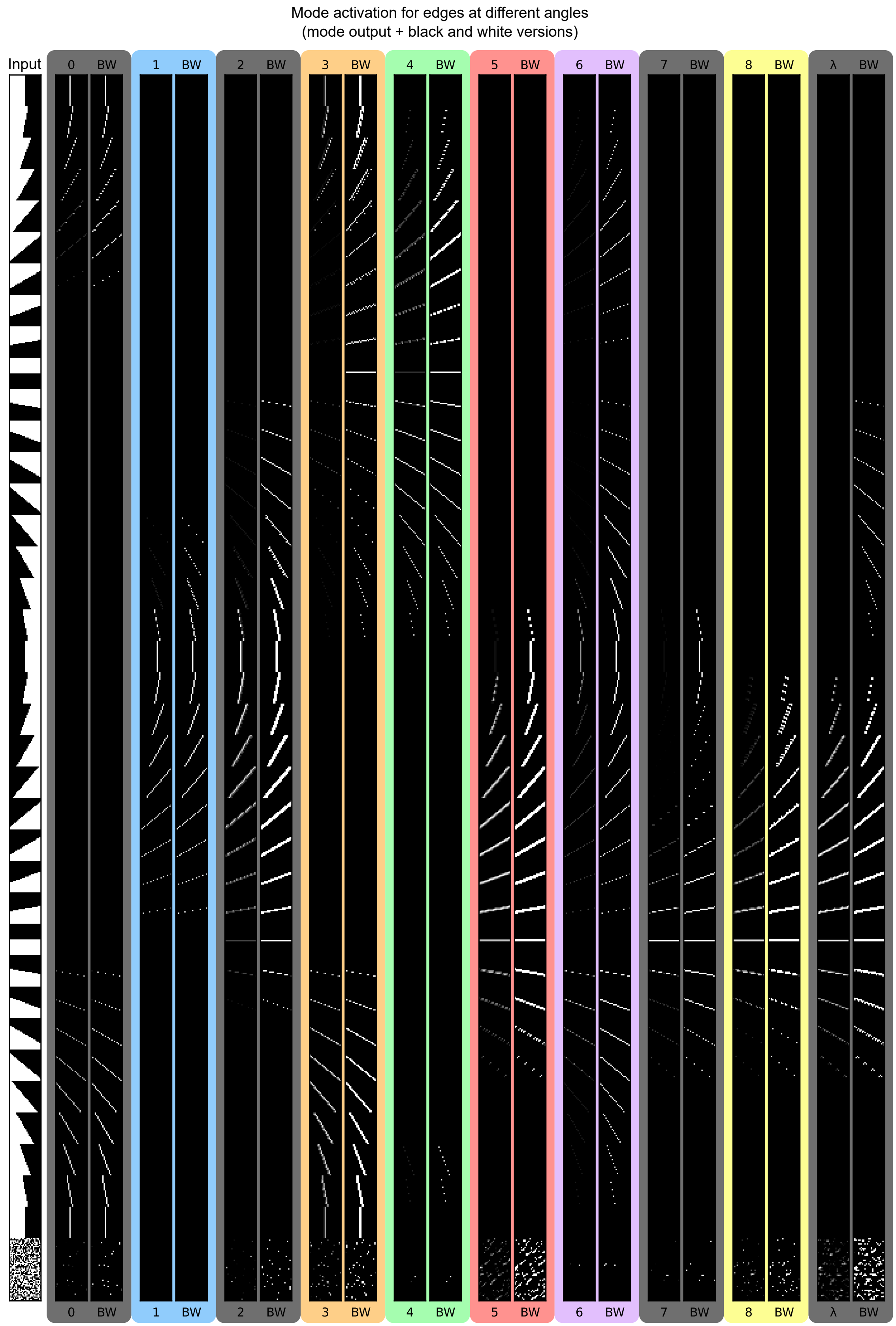

Supplementary Figure S10. Experimental feature maps for modes 0-9 of a range of rotated edges, and two white noise image inputs. The input images are shown in the left-most column. Each mode has two columns, an analogue-varying output produced by the mode-amplitude (left column for each mode, labelled with mode number) and a binarised black and white output produced by normalising the analogue output to 0 or maximum pixel intensity (right column for each mode, labelled BW.)

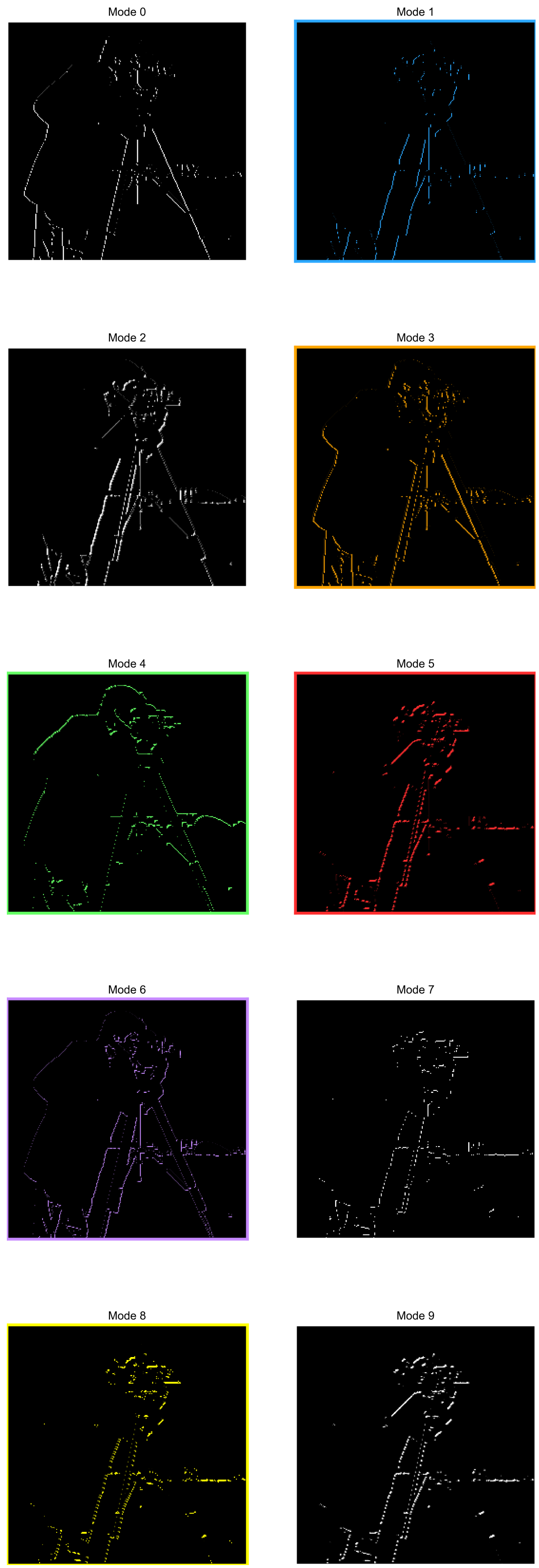

Supplementary Figure S11. Experimental feature maps of the 'cameraman' test image for modes 0-9. Additional feature maps are included compared to the six feature maps in Fig 1

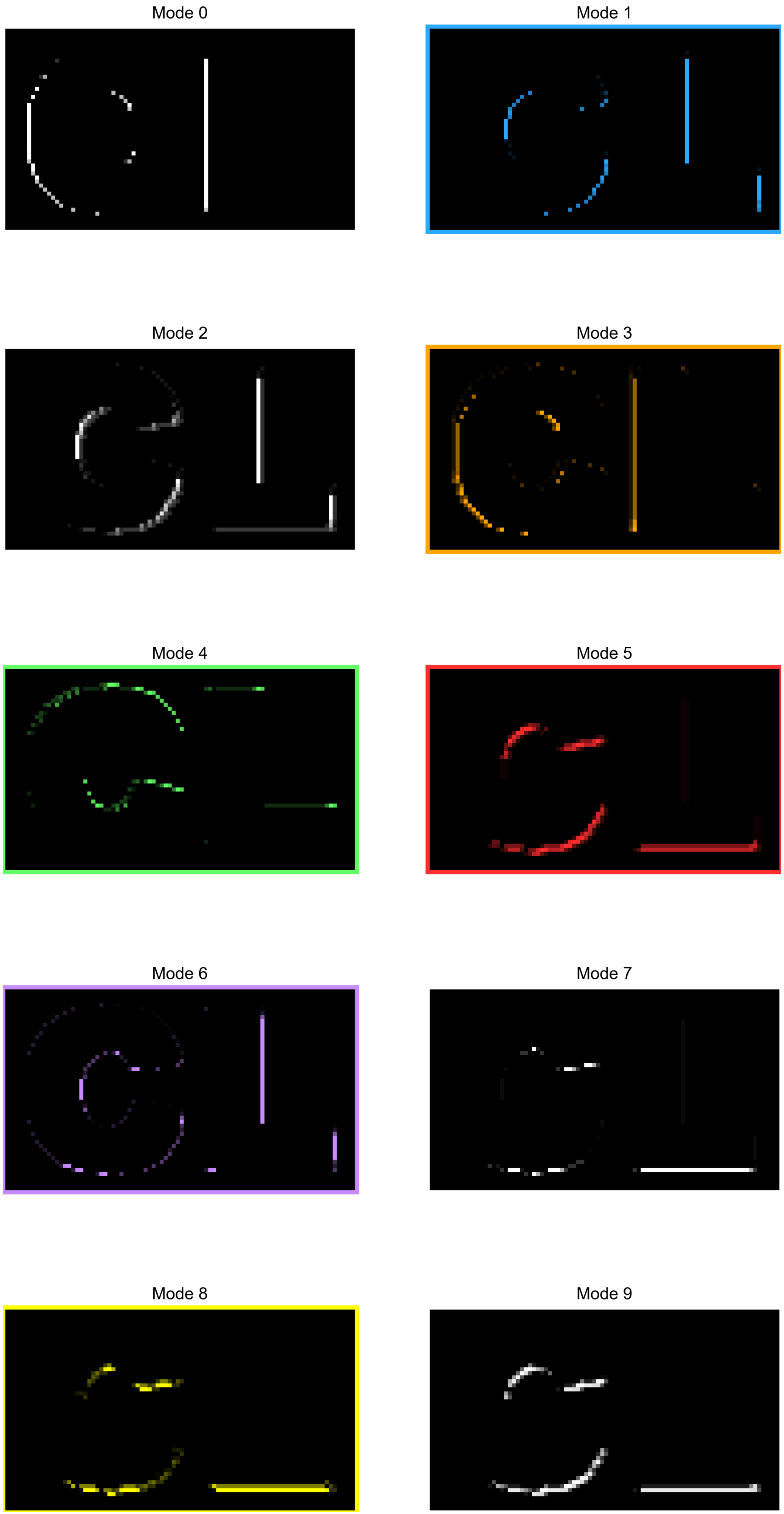

Supplementary Figure S12. Experimental feature maps of 'CL' test image for modes 0-9.

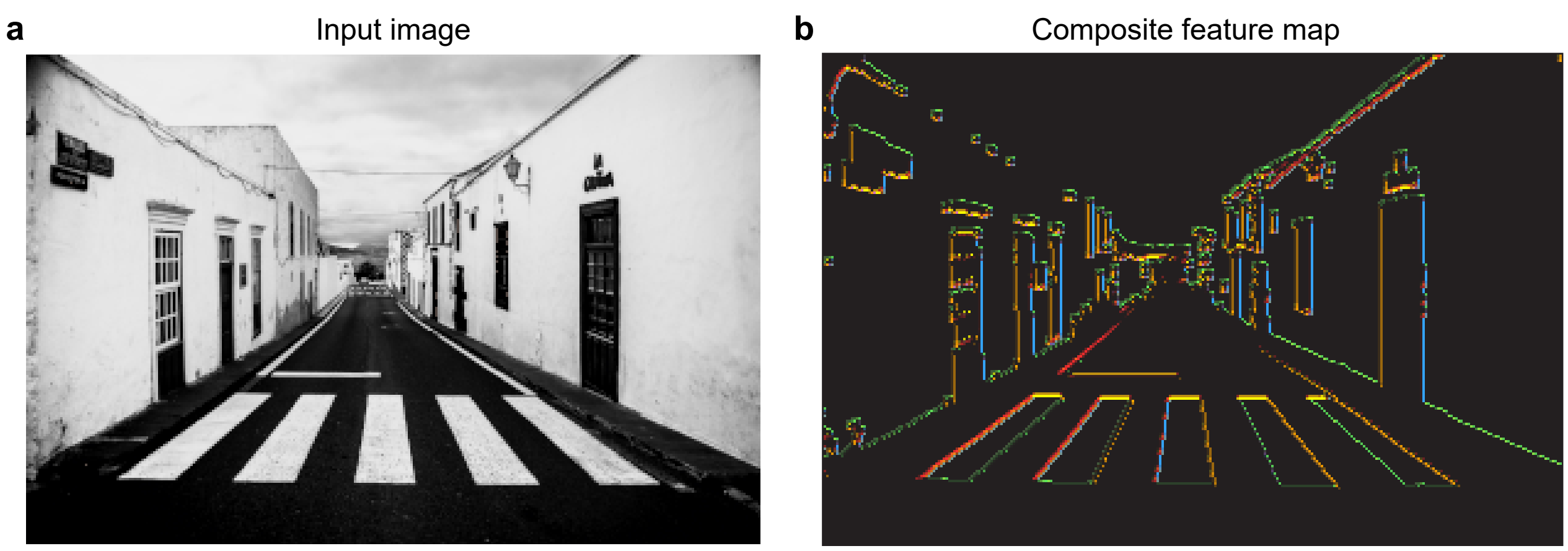

Supplementary Figure S13. 'Street' test image Input (A) and experimental composite feature map (B). License CC0 1.0 CC0 1.0

.

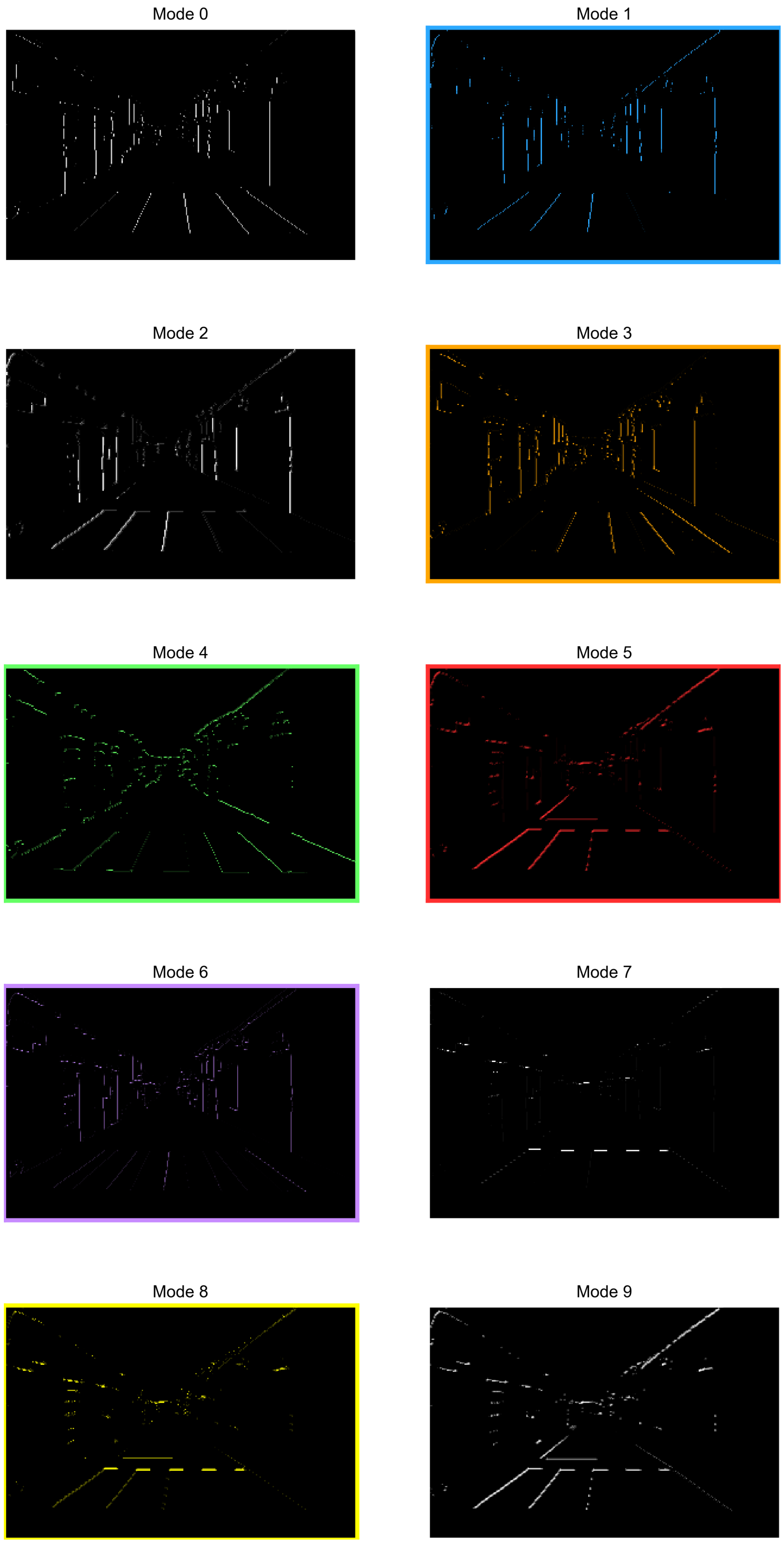

Supplementary Figure S14. Experimental feature maps of 'street' test image for modes 0-9. License CC0 1.0 CC0 1.0.

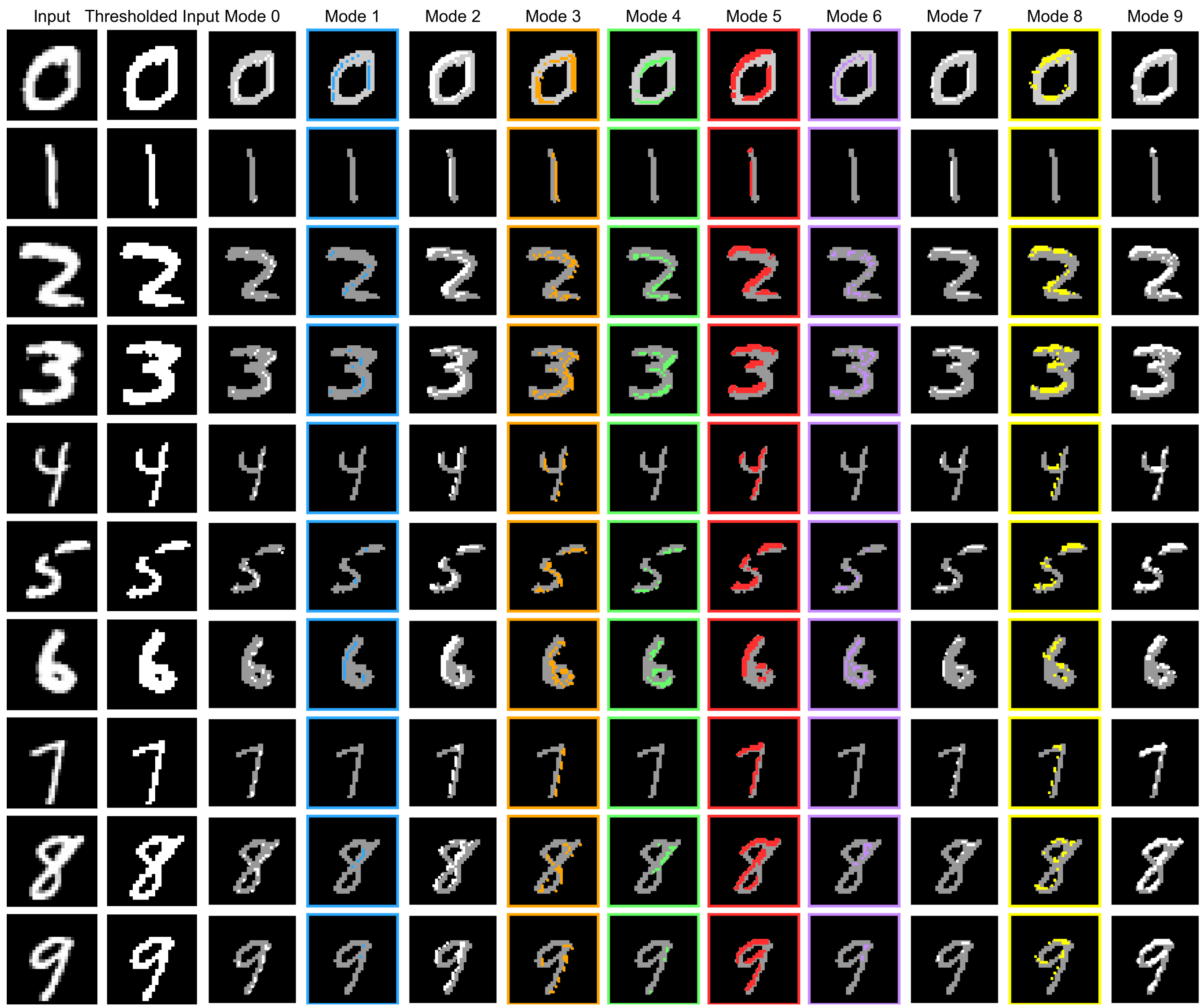

Supplementary Figure S15. Experimental feature maps of ten MNIST digits test images. One per digit class, for modes 0-9.

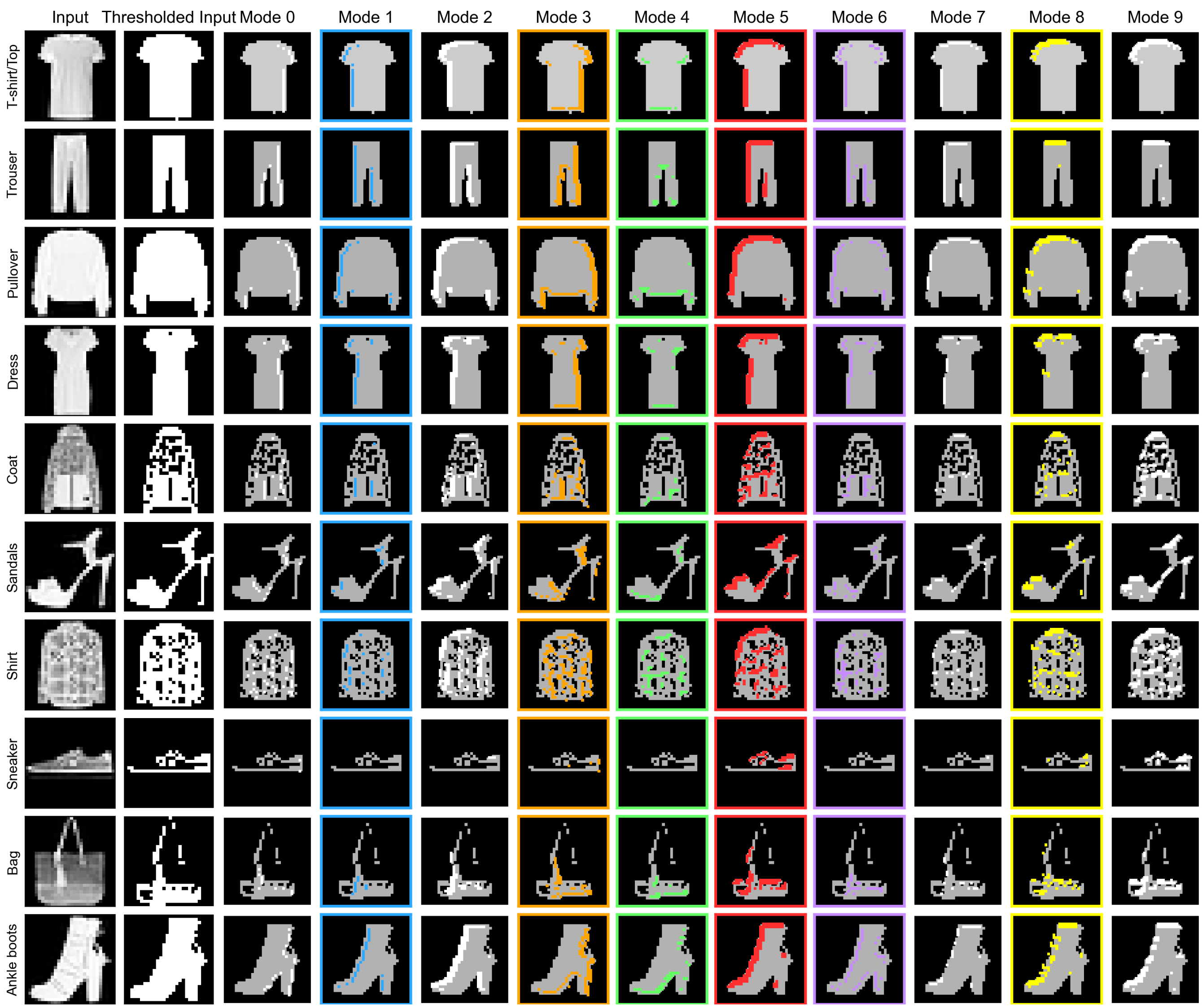

Supplementary Figure S16. Experimental feature maps of ten Fashion-MNIST test images. One feature map per object class, for modes 0-9.

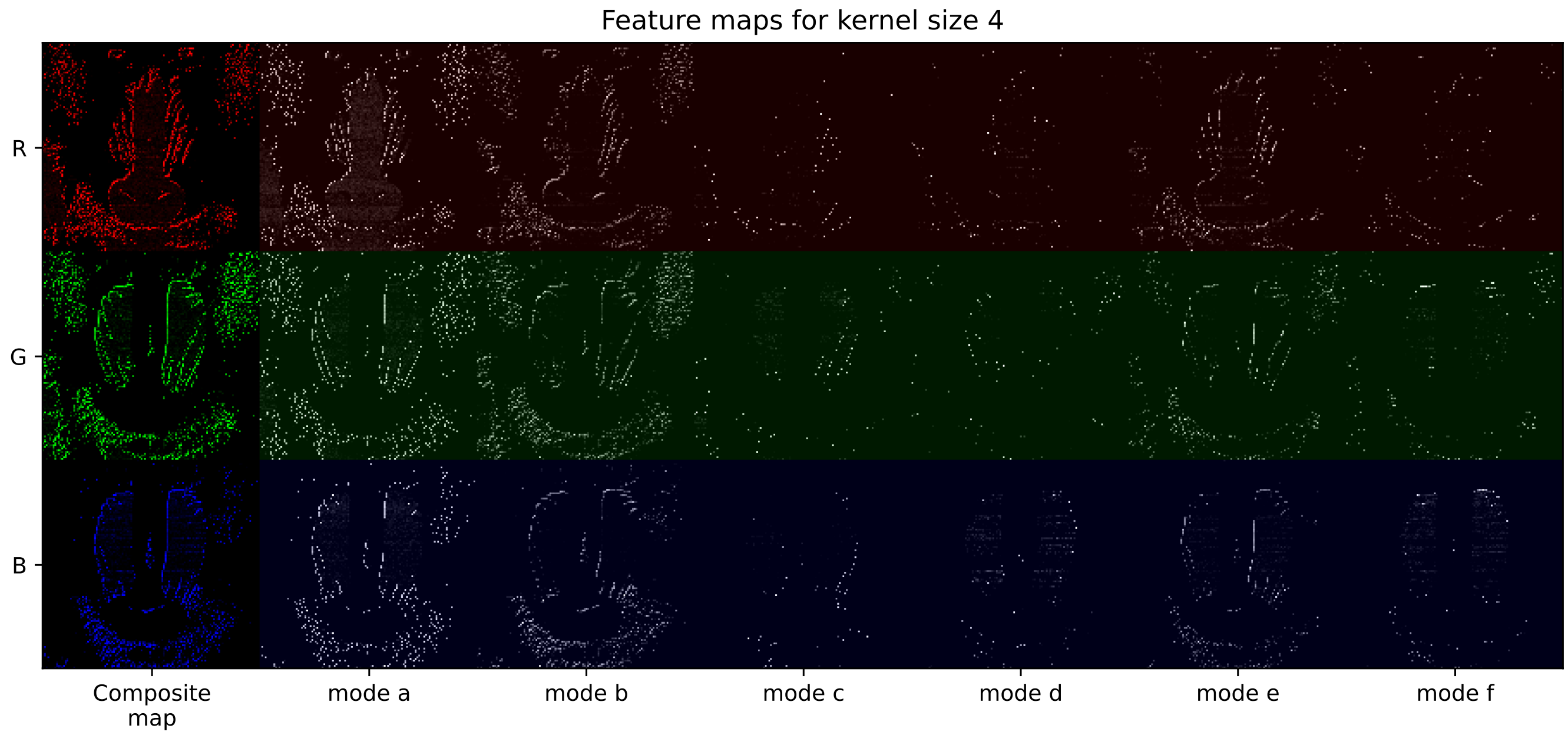

Supplementary Figure S17. Experimental feature maps of for the mandrill test image scanned with a 4x4 raster window. Separate scans were performed for the red, green, and blue image channels, but each scan uses the same modes for the feature detection.

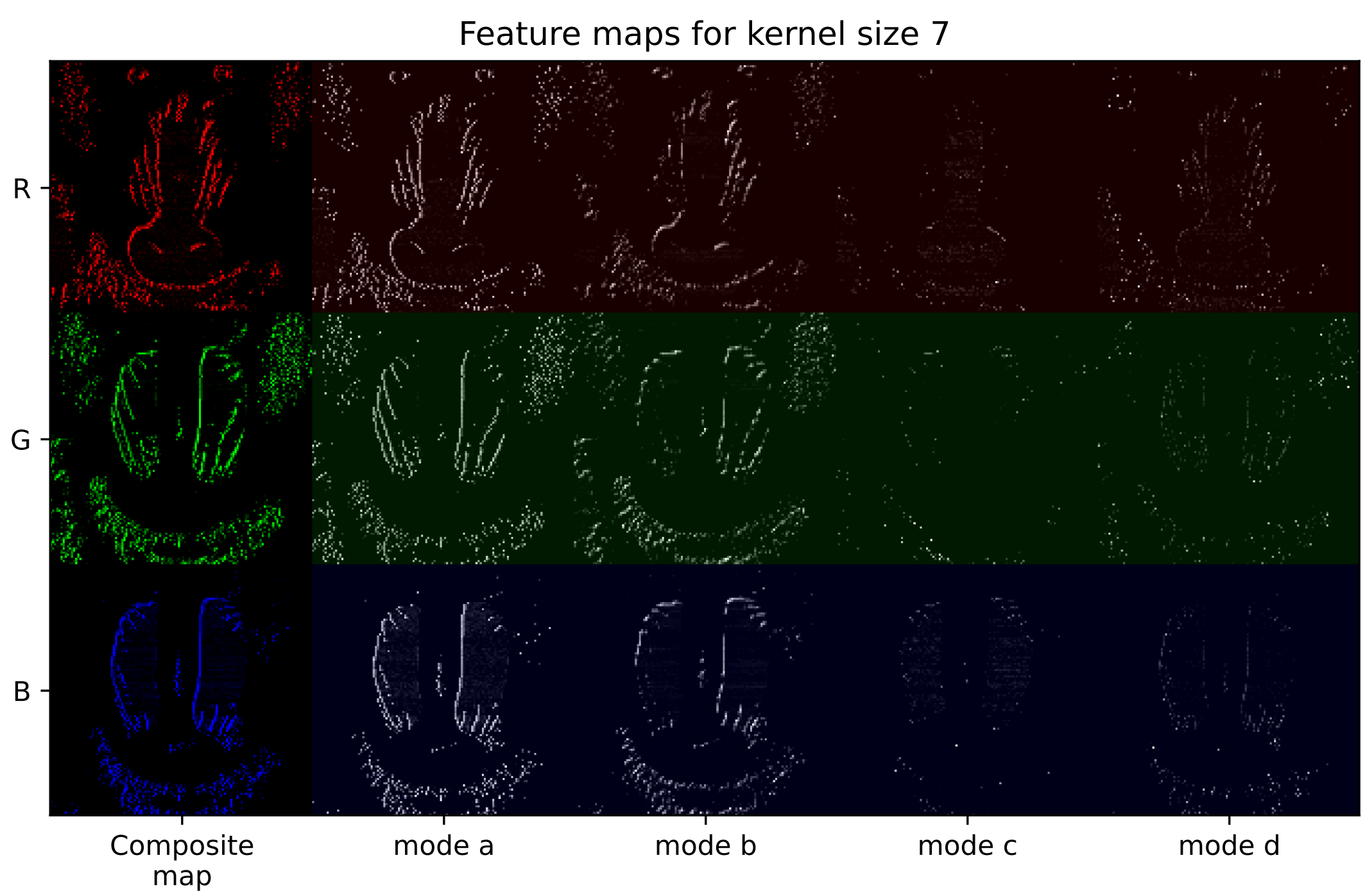

Supplementary Figure S18. Experimental feature maps of for the mandrill test image scanned with a 7x7 raster window. Separate scans were performed for the red, green, and blue image channels, but each scan uses the same modes for the feature detection.

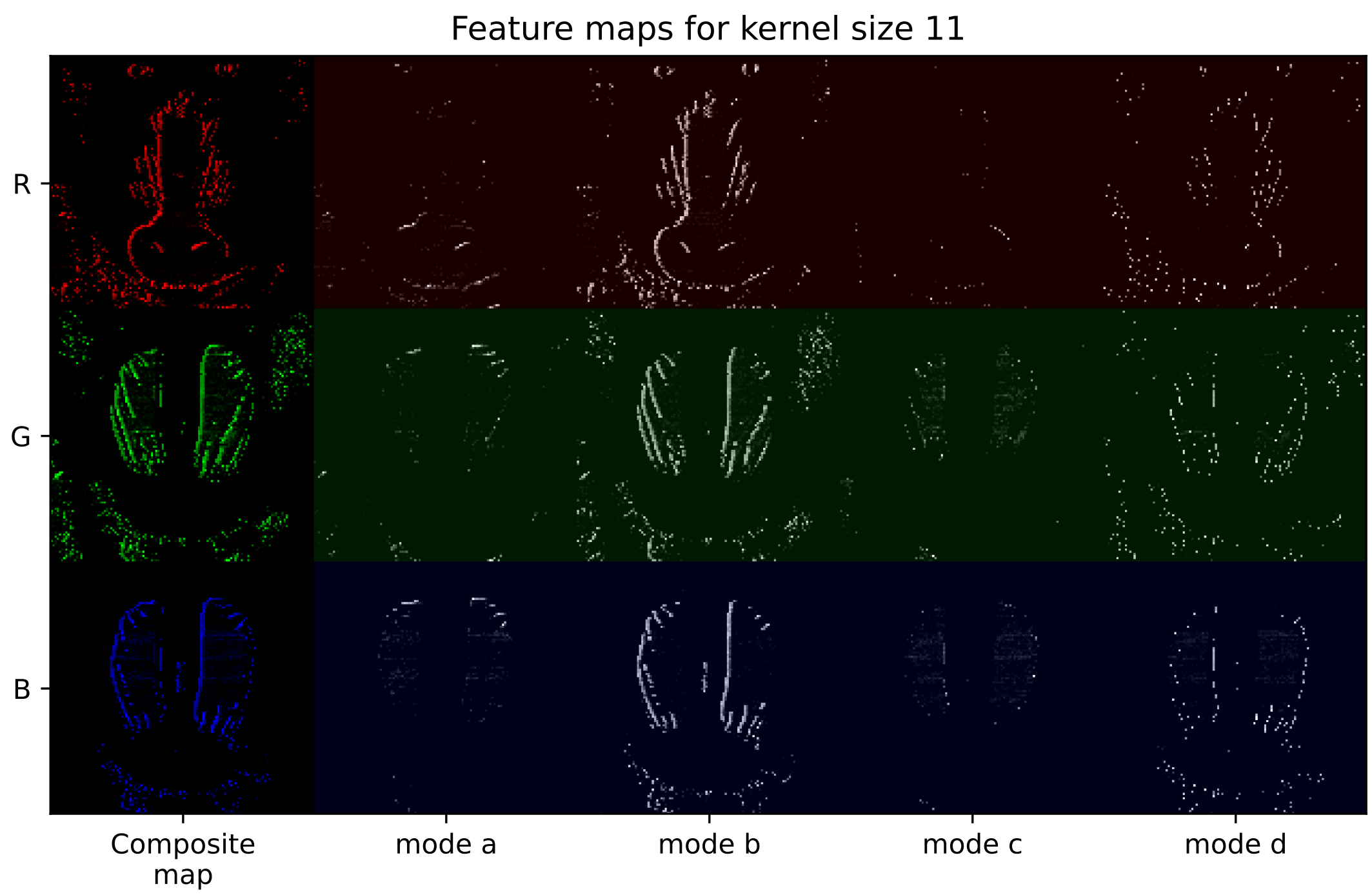

Supplementary Figure S19. Experimental feature maps for the mandrill test image scanned with a 11x11 raster window. Separate scans were performed for the red, green, and blue image channels, but each scan uses the same modes for the feature detection.

Least-squares fitting of kernel filters

As described in the main text and shown in Fig. 2I, we perform linear least-squares fitting between input images and experimentally measured mode amplitudes to fit effective linear kernel filters for the experimental modes performing feature detection. Results of this process are shown in Fig. S20. The fitting process uses a least-squares method to find the linear kernel filter weights which best reproduce the filter output (e.g. the lasing mode amplitude for our experimental case) for a given set of input images and filter outputs. As our input image set, we use the 65,536 (2^{16}) possible 4×4 black and white pixel images.

We plot the results of the fitting process for our ten experimental feature-detecting modes in the blue left-hand column of Fig. S20, titled 'experimental fits'. For the 65,536 input images we show the target output (eg. the experimental lasing mode amplitude) and residual fitting errors in the blue and orange traces respectively in the column titled 'mode output' next to the fitted kernels. Substantial residual errors are observed, with R^2 values of 0.628-0.946. This suggests that the linear fitting process may be unable to fully capture the feature detecting dynamics of the experimental system.

To test this assumption, we also performed the same fitting process on linear software kernel filters, initialising ten 4×4 filters with randomly generated weights. The random kernels are plotted in the left-hand orange column titled 'random kernels'. The results of the linear fitting process for these kernels are shown in the adjacent columns titled 'fit' (the kernel weights) and 'convolved with $4{\times}4$ images' (target and residual errors). Here, perfect R^2 scores of 1.0 are observed, as expected when performing a linear fit on a linear process. To test whether the poorer-quality linear fits to our experimental data are a result of experimental noise, we performed a further test where we added Gaussian noise to the software filter output. We used a noise amplitude of 10% relative to the maximum software kernel weights. Results of this test are shown in the two right-most columns. Lower R^2 of 0.978-0.988 are observed relative to the no-noise case, but still substantially higher than the fits to the experimental data.

Figure S21 shows the distribution of R^2 values for fits beyond the 10 modes highlighted as feature-detecting in the manuscript. For hyperspectral bins where noise overwhelms the signal, the R^2 value would naturally be low, so this analysis is constrained to modes that reach a maximum amplitude of at least 10% of the overall maximum for the 65,536 input images. The distribution shows that all analysed modes have R^2 values lower than 1, with a peak around 0.91 and a long tail towards 0.6. Some nonlinear behaviour is thus exhibited by the majority of modes.

A similar linear fitting study was performed for the simulated netSALT results, and Fig. S22 shows a set of linearly-fitted kernels for the netSALT modes shown in Fig. S4. The R^2 values seen are significantly lower than 1, indicating that the linear fit is unable to fully capture the feature detection process in the simulated network, just as with the experimental one. Negative weights are also observed, illustrating the suppressive effects of mode competition. 401 simulated lasing responses to $8{\times}8$ raster slices of the CL image from Fig. 2 E were used as the input for fitting, which is smaller than the 65,536 experimental samples, but spans the 64-dimensional input space.

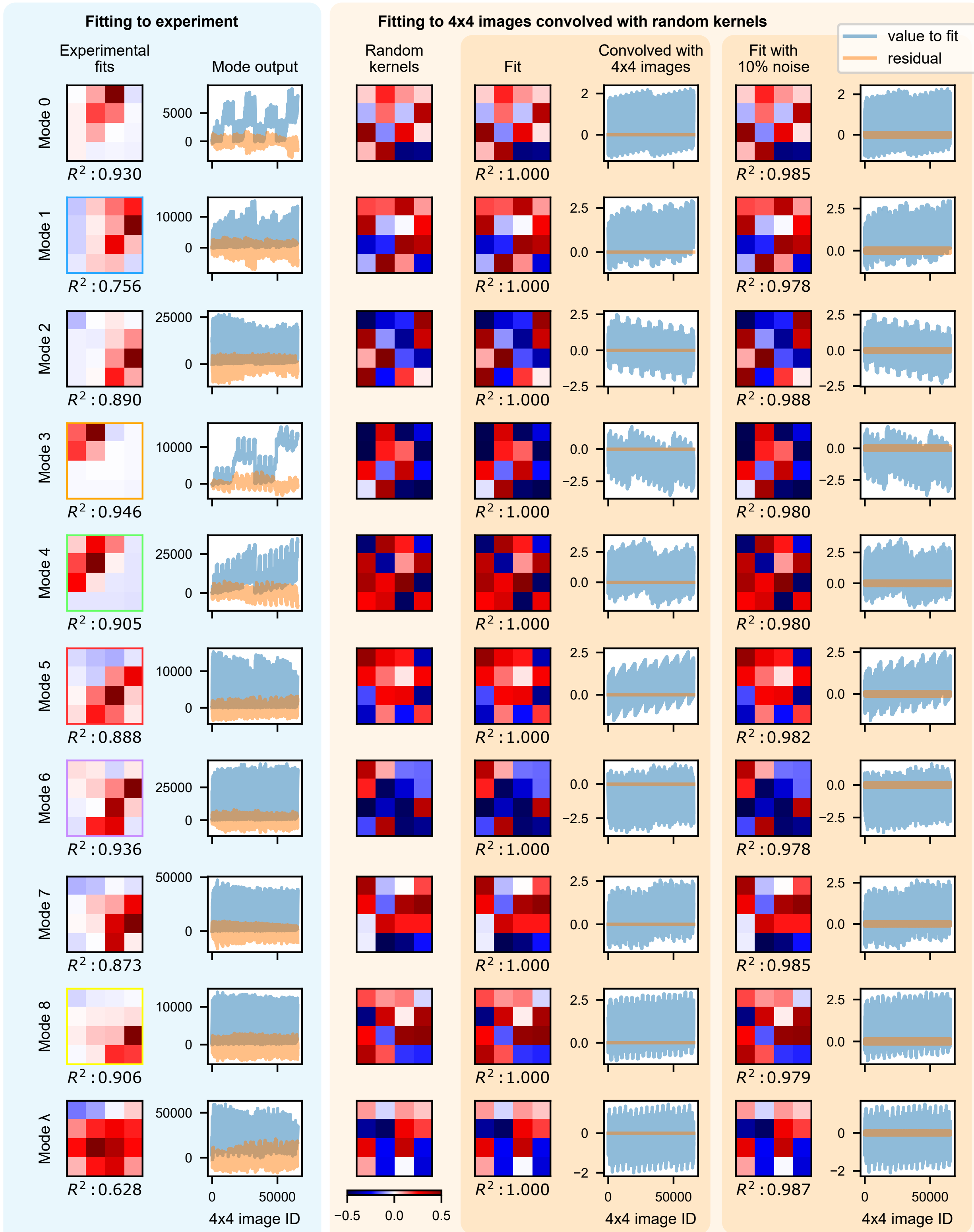

Supplementary Figure S20. Least-squares fitting of kernel filters. Left-hand blue column shows the results of the fitting process for the ten experimental feature detecting modes. R^2 values from 0.628-0.946 are found, suggesting aspects of the experimental feature detection functionality are not fully captured by the linear fitting. The orange and blue mode output traces show the target value to be fitted (blue) and the fitting error residual (orange) for the 65,536 (2^{16}) possible 4×4 pixel images. The central orange column shows the same process performed for randomly generated linear software kernel filters, showing perfect R^2 scores of 1.0. The right-hand column shows the same process performed where Gaussian noise of 10% amplitude relative to the maximum kernel weight is added, with reduced but still high R^2 scores of 0.978-0.988.

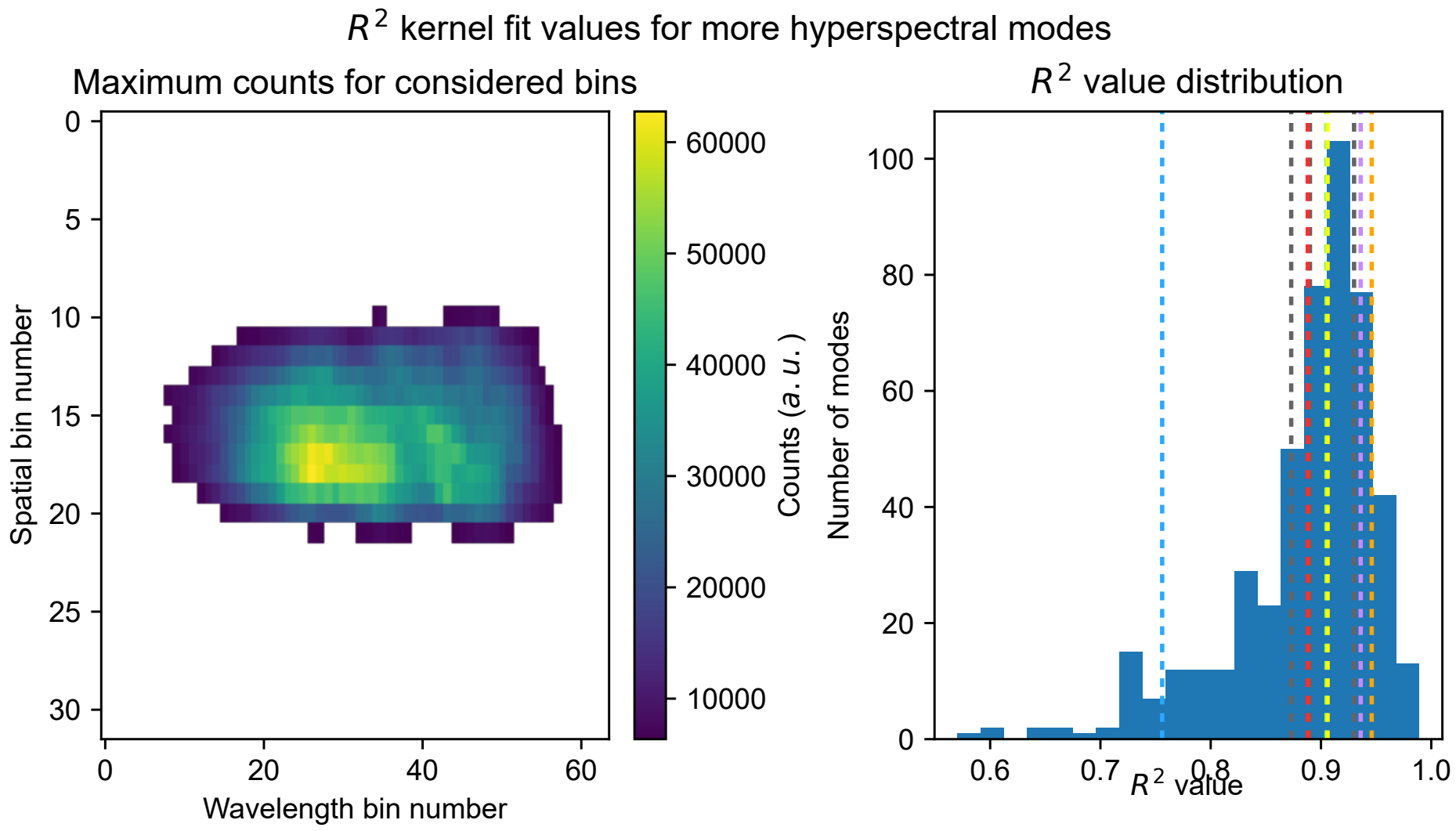

Supplementary Figure S21. Experimental R^2 fits for all binned hyperspectral modes that reach more than 10% of the maximum signal amplitude. Maximum counts for considered bins (left) and R^2 value distribution (right). Dashed lines show R^2 values for modes in Fig. S20.

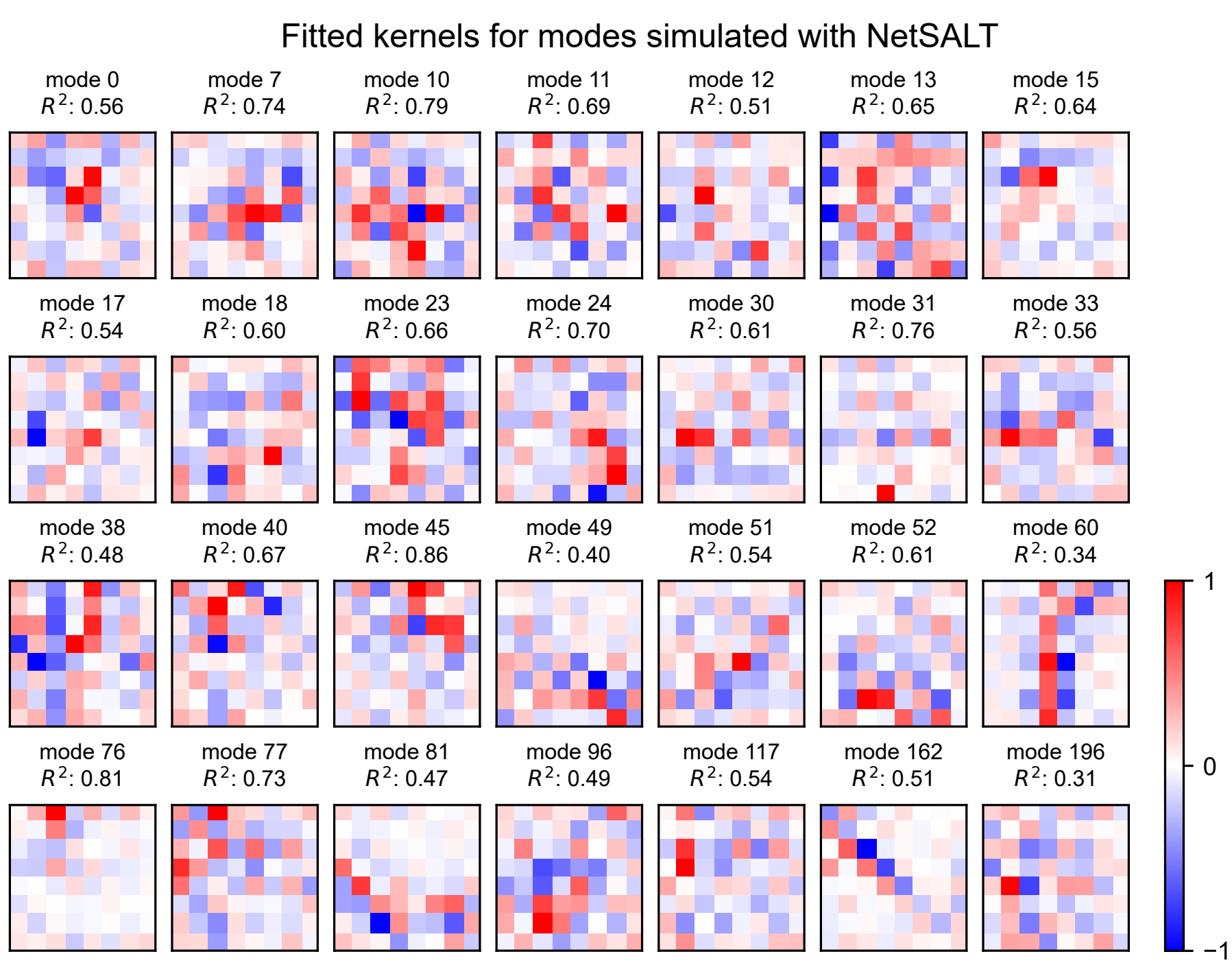

Supplementary Figure S22. Fitted kernels for netSALT-simulated modes shown in Fig. S4.

Quasi-isomorphic software benchmarks

To compare our system against a software network with similar architecture, we constructed a network with quasi-isomorphic structure to our experimental system. The quasi-isomorphic software benchmark comprises an initial convolutional layer with 10 4×4 convolutional filters, followed by a hidden layer of 30,000 neurons with randomised weights and ReLu nonlinear activation, with a final logistic regression layer. We compared our experimental system against two versions of this quasi-isomorphic software network: one with randomised kernel filter weights, and one with kernel filter weights obtained via fits to our experimental data as shown in Fig. 2F and S8, S9.

Figures S23 and S24 show the classification results of these tests for the MNIST digits and Fashion-MNIST tasks respectively. The experimental photonic system outperforms the quasi-isomorphic benchmark in both cases, with the software benchmark using the fitted experimental kernel weights performing better.

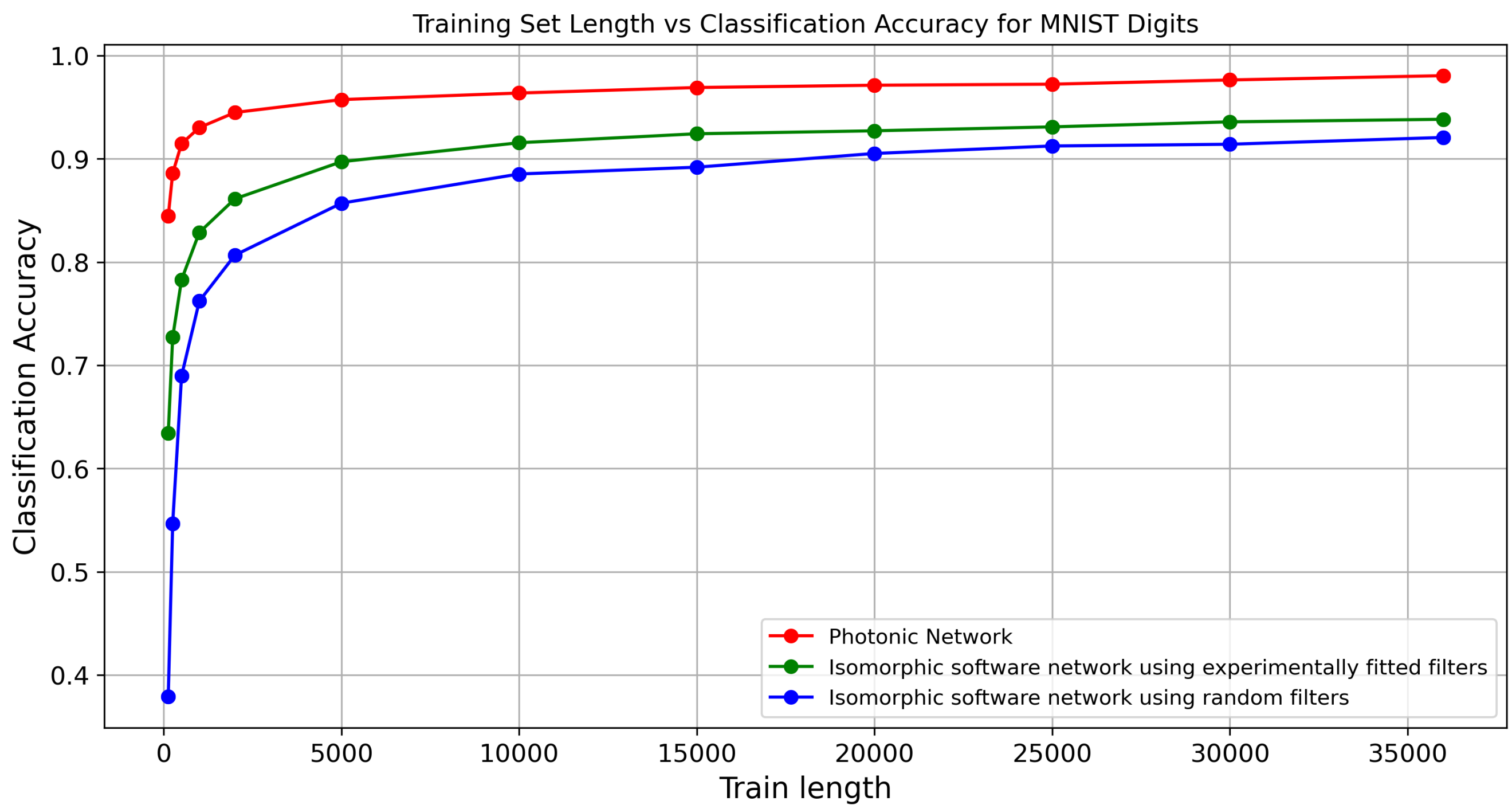

Supplementary Figure S23. MNIST digits classification results for the experimental photonic system. The results are for two versions of the quasi-isomorphic software benchmark, one with random kernel filter weights and one with kernel filter weights obtained via fits to our experimental data.

Speed, footprint, energy and scalability

Speed The experimental throughput for the results in this paper is up to 100 Hz. While the manuscript was in revision, we confirmed that our system can operate up to 1 kHz using an upgraded camera (Tucsen Aries 6510), recording lasing hyperspectra from the network. This was tested after collecting all the experimental data for this paper, and these faster results are not featured but will be further explored for subsequent works on our system. Beyond this 1 kHz rate, our next limit is our DMD which operates at 10 kHz. Commercial MHz DMDs are available, and DMD technology is currently rapidly advancing. For detection, commercially available Si or InGaAs photodiode arrays (e.g. Hamamatsu) run from $10^4 - 10^6$ Hz and are readily integrable with our scheme. Beyond this, the photonic dynamics of the system operate at 10-100 ps - going faster than this would require substantial re-engineering of the network material and system architecture, but these 10-100 GHz speeds are considerable. For reference, a modern NVIDIA 4090 GPU running a large CNN, ResNet or EfficientNet equivalent, takes about 1 ms per inference - e.g. 1 kHz operation, and consumes 600 W without considering cooling, RAM, memory buffering etc overheads.

Footprint Our current experimental optical system occupies an area of approximately 450 mm × 450 mm, with an external fs laser of size of 730 mm × 419 mm. The fs laser is guided into the optical system to pump the 150 µm diameter on-chip InP network laser, with the network providing all photonic nonlinear processing which drives computation. This is similar to other on-chip photonic computing schemes, such as large area vertical cavity surface emitting laser (LA-VCSEL) (∼20 um in diameter)[27] , with substantially reduced on-chip footprint relative to cross-bar arrays (>7 mm × 8 mm)[42] , and Mach–Zehnder interferometers (∼6 mm × 5.5 mm)[64] – which also currently require external laser sources. It is worth noting that the cross-bar

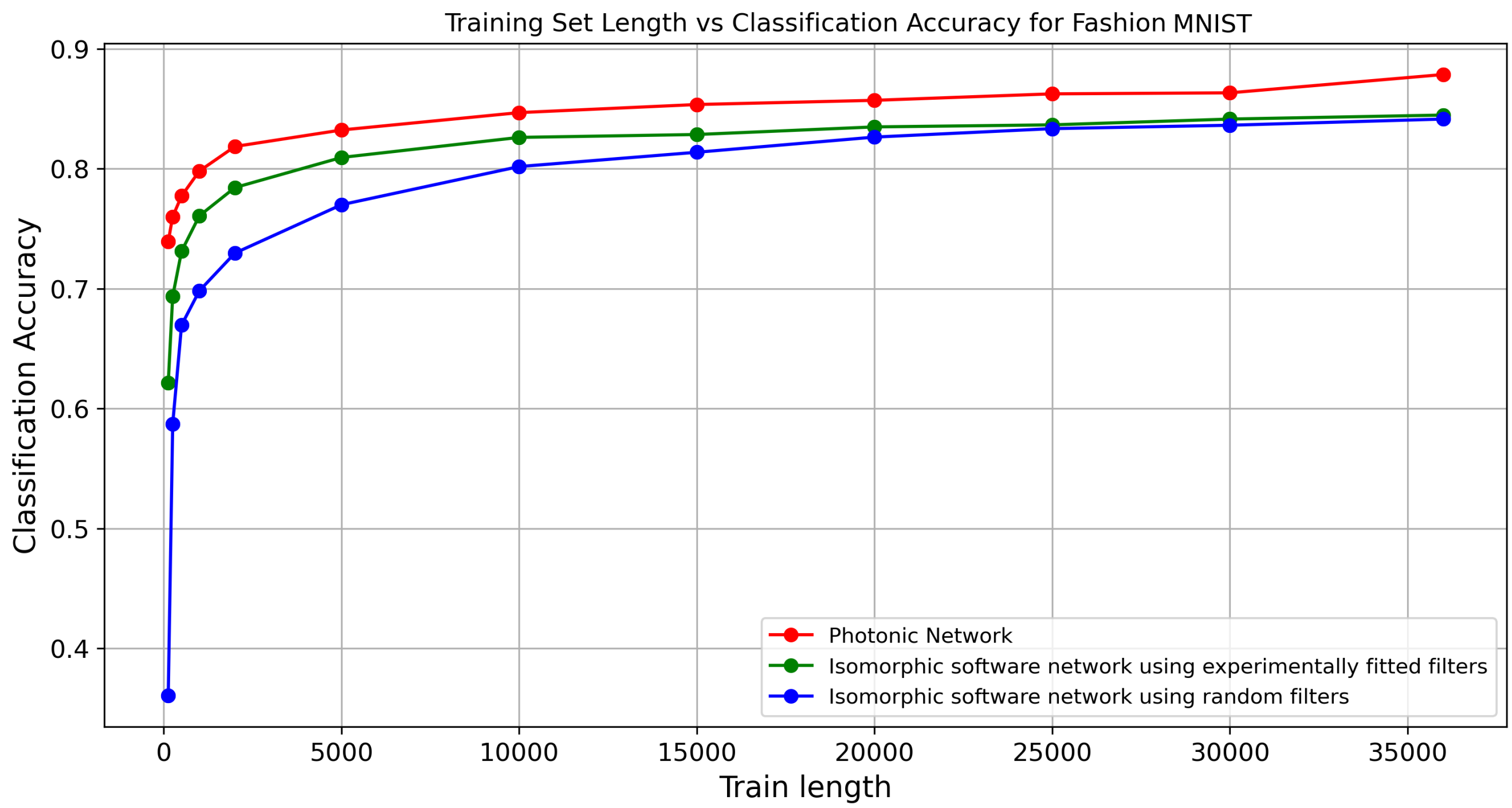

Supplementary Figure S24. Fashion MNIST classification results for the experimental photonic system. The results are for two versions of the quasi-isomorphic software benchmark, one with random kernel filter weights and one with kernel filter weights obtained via fits to our experimental data.

and Mach-Zehnder schemes provide degrees of on-chip programming. In future, on-chip programming (electro-optical, etc.) may be introduced to the network laser scheme.

To miniaturise the system in future, a more compact optical path design can be implemented to reach an off-chip optics footprint of <100 cm^2. As discussed in the Methods, the system can also be operated with a 80 ps diode pumped MOPA laser (180 mm $\times$ 90 mm), which is approximately 1/18th of the size of the fs laser. With further steps, future iterations could replace the off-chip pump laser with pixelated on-chip light sources, e.g., VCSEL and microLED arrays, to further reduce the footprint.

Energy Our experimental results for this paper were collected using a system with optical and electronic devices: A 200 fs laser source with an average power of 43 µW (pulse energy of 43 nJ) on the network (wall-plug power of 600 W), a DMD and a camera with operating power of 2.7 W and 5 W, respectively. Considering the 100 Hz operational speed of the system, the effective optical energy consumption is 0.43 µJ/frame, with the effective energy consumption of the whole system summed up to $\sim$77 mJ/frame. Since the digital regression layer at the end of our inference pipeline is lightweight, amounting to one matrix multiplication per illumination per class, we estimate the power used by the logistic regression step to be on the order of $\sim$0.5 µJ (single layer) - 5 µJ (two layer) per frame.

To put the pump energy in perspective, other photonic computing methods (with similar electronic system setup and off-chip power consumption), e.g., multimode fibre takes 8.7 µJ/frame[17], LA-VCSEL takes 27.7 µJ/frame[27], and diffractive media takes 12.5 mJ/frame[65].

Improving the efficiency of the pump laser will allow us to greatly improve power and energy consumption. To this end, we have confirmed during revision of the manuscript that a much lower power (10.5 W wall-plug power) 80 picosecond pulse pump laser (Picophotonics CP32, 532 nm) can drive our network to lase, with experimental confirmation shown in supplementary Fig. S3. Using this pump laser brings our wall-plug power consumption to 16.2 W, which bodes well for future iterations of our scheme using lower power laser and faster kHz throughput.

To put these numbers in perspective, an NVIDIA 4090 GPU consumes 600 W. For the BreaKHis biomedical task considered in this paper, a 4090 GPU running EfficientNetV2-B0 required 16 hours of runtime with 20$\times$ data augmentation (e.g. artificially making 20 augmented/distorted/rotated copies of each training image) in order to match the accuracy performance of our system, while our system took 4 minutes, including optical experiments and logistic regression training. This is of course a limited use-case, relatively hard vision tasks with small training sets, and for larger dataset machine learning tasks with many 10s-100s of thousands of training examples our system underperforms relative to conventional large modern software neural networks. However, these relatively hard, data-restricted tasks are highly relevant for the edge-AI use cases where neuromorphic computing may have a lot to offer. We envision that in future iterations of our scheme, this range of tasks where

we offer competitive performance will be substantially expanded by refinements to the physical system, optical experimental scheme, and training approach.

Beyond using free space pulsed lasers, the system should in future be compatible with on-chip pixelated light sources e.g. micro LED arrays and vertical cavity surface emitting lasers (VCSELs) which have far higher energy-efficiency than free-space pulsed lasers, up to 40-50% efficiency. This would have the added benefit of removing the requirement for a DMD and greatly reducing the system footprint.

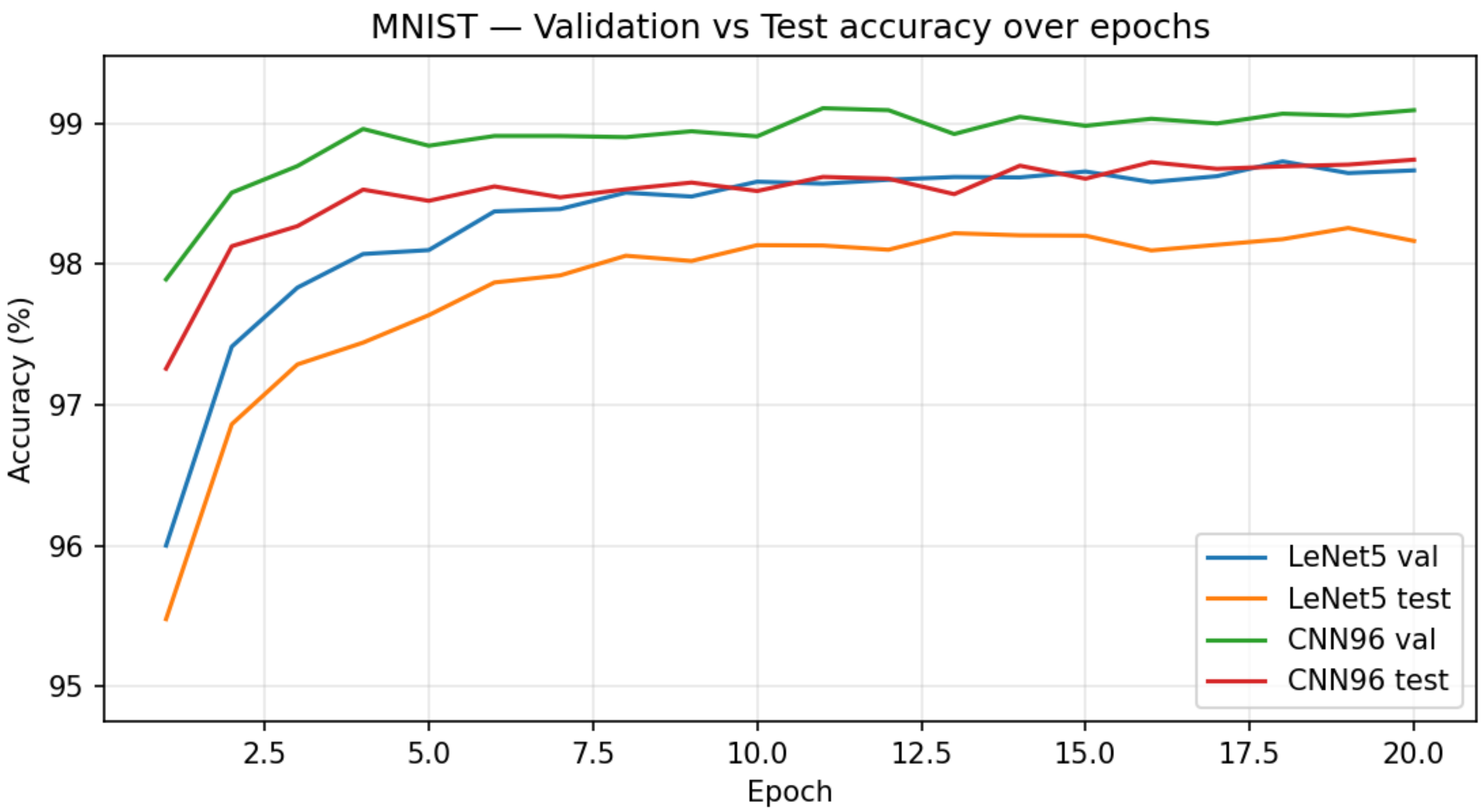

Supplementary Figure S25. Validation versus test accuracy on MNIST for the 'LeNet5' and '96-Filter CNN' software CNN baselines over training epochs. The models converge well with stable plateaus observed.

REFERENCES

1. D. Marković, A. Mizrahi, D. Querlioz, J. Grollier, Physics for neuromorphic computing. *Nat. Rev. Phys.* **2**, 499–510 (2020).

2. P. L. McMahon, The physics of optical computing. *Nat. Rev. Phys.* **5**, 717–734 (2023).

3. B. J. Shastri, A. N. Tait, T. Ferreira de Lima, W. H. P. Pernice, H. Bhaskaran, C. D. Wright, P. R. Prucnal, Photonics for artificial intelligence and neuromorphic computing. *Nat. Photonics* **15**, 102–114 (2021).

4. Y. LeCun, Y. L. Cun, L. D. Jackel, L. Bottou, C. Cortes, J. S. Denker, H. Drucker, I. Guyon, U. A. Müller, E. Säckinger, P. Simard, V. Vapnik, "Learning algorithms for classification: A comparison on handwritten digit recognition" in *Neural Networks: The Statistical Mechanics Perspective*, World Scientific Publishing (1995), vol. 261, pp. 2.

5. K. O'Shea, R. Nash, An introduction to convolutional neural networks. arXiv:1511.08458 [cs. NE] (2015).

6. A. Vaswani, N. Shazeer, N. Parmar, J. Uszkoreit, L. Jones, A. N. Gomez, L. Kaiser, I. Polosukhin, "Attention is all you need" in *Advances in Neural Information Processing Systems 30* (*NIPS*, 2017).

7. S. Khan, M. Naseer, M. Hayat, S. W. Zamir, F. S. Khan, M. Shah, Transformers in vision: A survey. *ACM Comput. Surv.* **54**, 1–41 (2022).

8. Z. Li, F. Liu, W. Yang, S. Peng, J. Zhou, A survey of convolutional neural networks: Analysis, applications, and prospects. *IEEE Trans. Neural Netw. Learn. Syst.* **33**, 6999–7019 (2022).

9. J. Gu, Z. Wang, J. Kuen, L. Ma, A. Shahroudy, B. Shuai, T. Liu, X. Wang, G. Wang, J. Cai, T. Chen, Recent advances in convolutional neural networks. *Pattern Recognit.* **77**, 354–377 (2018).

10. Y. Song, T. Wang, P. Cai, S. K. Mondal, J. P. Sahoo, A comprehensive survey of few-shot learning: Evolution, applications, challenges, and opportunities. *ACM Comput. Surv.* **55**, 1–40 (2023).

11. I. Y. Tyukin, A. N. Gorban, M. H. Alkhudaydi, Q. Zhou, “Demystification of few-shot and one-shot learning” in *2021 International Joint Conference on Neural Networks (IJCNN)* (IEEE, 2021), pp. 1–7.

12. A. Momeni, B. Rahmani, B. Scellier, L. G. Wright, P. L. McMahon, C. C. Wanjura, Y. Li, A. Skalli, N. G. Berloff, T. Onodera, I. Oguz, F. Morichetti, P. del Hougne, M. le Gallo, A. Sebastian, A. Mirhoseini, C. Zhang, D. Marković, D. Brunner, C. Moser, S. Gigan, F. Marquardt, A. Ozcan, J. Grollier, A. J. Liu, D. Psaltis, A. Alù, R. Fleury, Training of physical neural networks. *Nature* **645**, 53–61 (2025).

13. N. Farmakidis, B. Dong, H. Bhaskaran, Integrated photonic neuromorphic computing: Opportunities and challenges. *Nat. Rev. Electr. Eng.* **1**, 358–373 (2024).

14. F. Jebali, A. Majumdar, C. Turck, K. E. Harabi, M. C. Faye, E. Muhr, J. P. Walder, O. Bilousov, A. Michaud, E. Vianello, T. Hirtzlin, F. Andrieu, M. Bocquet, S. Collin, D. Querlioz, J. M. Portal, Powering AI at the edge: A robust, memristor-based binarized neural network with near-memory computing and miniaturized solar cell. *Nat. Commun.* **15**, 741 (2024).

15. D. A. Allwood, M. O. A. Ellis, D. Griffin, T. J. Hayward, L. Manneschi, M. F. K. H. Musameh, S. O'Keefe, S. Stepney, C. Swindells, M. A. Trefzer, E. Vasilaki, G. Venkat, I. Vidamour, C. Wringe, A perspective on physical reservoir computing with nanomagnetic devices. *Appl. Phys. Lett.* **122**, 040501 (2023).

16. L. Manneschi, I. T. Vidamour, K. D. Stenning, C. Swindells, G. Venkat, D. Griffin, L. Gui, D. Sonawala, D. Donskikh, D. Hariga, E. Donati, S. Stepney, W. R. Branford, J. C. Gartside, T. J. Hayward, M. O. A. Ellis, E. Vasilaki, Noise-aware training of neuromorphic dynamic device networks. *Nat. Commun.* **16**, 9192 (2025).

17. I. Oguz, J. L. Hsieh, N. U. Dinc, U. Teğin, M. Yildirim, C. Gigli, C. Moser, D. Psaltis, Programming nonlinear propagation for efficient optical learning machines. *Adv. Photonics* **6**, 016002 (2024).

18. D. Kerschensteiner, Feature detection by retinal ganglion cells. *Annu. Rev. Vis. Sci.* **8**, 135–169 (2022).

19. R. H. Masland, The neuronal organization of the retina. *Neuron* **76**, 266–280 (2012).

20. W. B. Thoreson, S. C. Mangel, Lateral interactions in the outer retina. *Prog. Retin. Eye Res.* **31**, 407–441 (2012).

21. F. S. Werblin, The retinal hypercircuit: A repeating synaptic interactive motif underlying visual function. *J. Physiol.* **589**, 3691–3702 (2011).

22. A. L. Fairhall, C. A. Burlingame, R. Narasimhan, R. A. Harris, J. L. Puchalla, M. J. Berry II, Selectivity for multiple stimulus features in retinal ganglion cells. *J. Neurophysiol.* **96**, 2724–2738 (2006).

23. N. Perez-Nieves, V. C. Leung, P. L. Dragotti, D. F. Goodman, Neural heterogeneity promotes robust learning. *Nat. Commun.* **12**, 5791 (2021).

24. Y. Shen, N. C. Harris, S. Skirlo, M. Prabhu, T. Baehr-Jones, M. Hochberg, X. Sun, S. Zhao, H. Larochelle, D. Englund, M. Soljačić, Deep learning with coherent nanophotonic circuits. *Nat. Photonics* **11**, 441–446 (2017).

25. N. Savage, Light could lower AI's appetite for power. *Nat. Nanotechnol.* **21**, 6–8 (2026).

26. T. Wang, M. M. Sohoni, L. G. Wright, M. M. Stein, S. Y. Ma, T. Onodera, M. G. Anderson, P. L. McMahon, Image sensing with multilayer nonlinear optical neural networks. *Nat. Photonics* **17**, 408–415 (2023).

27. X. Porte, A. Skalli, N. Haghighi, S. Reitzenstein, J. A. Lott, D. Brunner, A complete, parallel and autonomous photonic neural network in a semiconductor multimode laser. *J. Phys. Photonics* **3**, 024017 (2021).

28. A. Skalli, S. Sunada, M. Goldmann, M. Gebski, S. Reitzenstein, J. A. Lott, T. Czyszanowski, D. Brunner, Model-free front-to-end training of a large high performance laser neural network. arXiv:2503.16943 [cs.LG] (2025).

29. D. Saxena, A. Fischer, J. Dranczewski, W. K. Ng, N. V. Trivino, H. Schmid, T. V. Raziman, A. Arnaudon, M. Barahona, O. Hess, K. Moselund, R. Sapienza, Designed semiconductor network random lasers. *Laser Photonics Rev.* **19**, 2400623 (2025).

30. J. Dranczewski, A. Fischer, P. Tiwari, M. Scherrer, D. Saxena, H. Schmid, R. Sapienza, K. Moselund, Plasma etching for fabrication of complex nanophotonic lasers from bonded InP semiconductor layers. *Micro Nano Eng.* **19**, 100196 (2023).

31. H. Cao, R. Chriki, S. Bittner, A. A. Friesem, N. Davidson, Complex lasers with controllable coherence. *Nat. Rev. Phys.* **1**, 156–168 (2019).

32. R. Sapienza, Controlling random lasing action. *Nat. Phys.* **18**, 976–979 (2022).

33. D. S. Wiersma, The physics and applications of random lasers. *Nat. Phys.* **4**, 359–367 (2008).

34. D. Saxena, A. Arnaudon, O. Cipolato, M. Gaio, A. Quentel, S. Yaliraki, D. Pisignano, A. Camposeo, M. Barahona, R. Sapienza, Sensitivity and spectral control of network lasers. *Nat. Commun.* **13**, 6493 (2022).

35. A. Cerjan, B. Redding, L. Ge, S. F. Liew, H. Cao, A. Douglas Stone, Controlling mode competition by tailoring the spatial pump distribution in a laser: A resonance-based approach. *Opt. Express* **24**, 26006–26015 (2016).

36. K. L. Van der Molen, R. W. Tjerkstra, A. P. Mosk, A. Lagendijk, Spatial extent of random laser modes. *Phys. Rev. Lett.* **98**, 143901 (2007).

37. N. Bachelard, S. Gigan, X. Noblin, P. Sebbah, Adaptive pumping for spectral control of random lasers. *Nat. Phys.* **10**, 426–431 (2014).

38. M. Gaio, D. Saxena, J. Bertolotti, D. Pisignano, A. Camposeo, R. Sapienza, A nanophotonic laser on a graph. *Nat. Commun.* **10**, 226 (2019).

39. A. Dosovitskiy. An image is worth 16x16 words: Transformers for image recognition at scale. arXiv:2010.11929 [cs.CV] (2020).

40. J. Chen, X. Ran, Deep learning with edge computing: A review. *Proc. IEEE* **107**, 1655–1674 (2019).

41. Y. Zeng, R. Zhou, L. Jiao, Z. Han, J. Yu, Y. Ma, "Efficient online DNN inference with continuous learning in edge computing" in *2024 IEEE/ACM 32nd International Symposium on Quality of Service (IWQoS)* (IEEE, 2024), pp. 1–10.

42. J. Feldmann, N. Youngblood, M. Karpov, H. Gehring, X. Li, M. Stappers, M. le Gallo, X. Fu, A. Lukashchuk, A. S. Raja, J. Liu, C. D. Wright, A. Sebastian, T. J. Kippenberg, W. H. P. Pernice, H. Bhaskaran, Parallel convolutional processing using an integrated photonic tensor core. *Nature* **589**, 52–58 (2021).

43. X. Xu, M. Tan, B. Corcoran, J. Wu, A. Boes, T. G. Nguyen, S. T. Chu, B. E. Little, D. G. Hicks, R. Morandotti, A. Mitchell, D. J. Moss, 11 Tops photonic convolutional accelerator for optical neural networks. *Nature* **589**, 44–51 (2021).

44. P. Yao, H. Wu, B. Gao, J. Tang, Q. Zhang, W. Zhang, J. J. Yang, H. Qian, Fully hardware-implemented memristor convolutional neural network. *Nature* **577**, 641–646 (2020).

45. H. Zheng, Q. Liu, I. I. Kravchenko, X. Zhang, Y. Huo, J. G. Valentine, Multichannel meta-imagers for accelerating machine vision. *Nat. Nanotechnol.* **19**, 471–478 (2024).

46. R. Sapienza, Determining random lasing action. *Nat. Rev. Phys.* **1**, 690–695 (2019).

47. H. E. Türeci, L. Ge, S. Rotter, A. D. Stone, Strong interactions in multimode random lasers. *Science* **320**, 643–646 (2008).

48. L. Ge, Y. D. Chong, A. D. Stone, Steady-state ab initio laser theory: Generalizations and analytic results. *Phys. Rev. A* **82**, 063824 (2010).

49. G. Levkine, Test images Collections, www.hlevkin.com/hlevkin/06testimages.htm.

50. University of Southern California, USC-SIPI Image Database—Mandrill Image, http://sipi.usc.edu/database/database.php?volume=misc.

51. M. Tan, Q. Le, “Efficientnet: Rethinking model scaling for convolutional neural networks” in *International Conference on Machine Learning* (PMLR, 2019), pp. 6105–6114.

52. M. Tan, Q. Le, “Efficientnetv2: Smaller models and faster training” in *International Conference on Machine Learning* (PMLR, 2021), pp. 10096–10106.

53. F. A. Spanhol, L. S. Oliveira, C. Petitjean, L. Heutte, A dataset for breast cancer histopathological image classification. *IEEE Trans. Biomed. Eng.* **63**, 1455–1462 (2016).

54. G.-B. Huang, Q.-Y. Zhu, C.-K. Siew, Extreme learning machine: Theory and applications. *Neurocomputing* **70**, 489–501 (2006).

55. K. Han, Y. Wang, H. Chen, X. Chen, J. Guo, Z. Liu, Y. Tang, A. Xiao, C. Xu, Y. Xu, Z. Yang, Y. Zhang, D. Tao, A survey on vision transformer. *IEEE Trans. Pattern Anal. Mach. Intell.* **45**, 87–110 (2023).

56. M. R. Alom, F. A. Farid, M. A. Rahaman, A. Rahman, T. Debnath, A. S. M. Miah, S. Mansor, An explainable AI-driven deep neural network for accurate breast cancer detection from histopathological and ultrasound images. *Sci. Rep.* **15**, 17531 (2025).

57. P. Tschandl, C. Rinner, Z. Apalla, G. Argenziano, N. Codella, A. Halpern, M. Janda, A. Lallas, C. Longo, J. Malvehy, J. Paoli, S. Puig, C. Rosendahl, H. P. Soyer, I. Zalaudek, H. Kittler, Human–computer collaboration for skin cancer recognition. *Nat. Med.* **26**, 1229–1234 (2020).

58. P. Tschandl, C. Rosendahl, H. Kittler, The HAM10000 dataset, a large collection of multi-source dermatoscopic images of common pigmented skin lesions. *Sci. Data* **5**, 180161 (2018).

59. K. D. Stenning, J. C. Gartside, L. Manneschi, C. T. S. Cheung, T. Chen, A. Vanstone, J. Love, H. Holder, F. Caravelli, H. Kurebayashi, K. Everschor-Sitte, E. Vasilaki, W. R. Branford, Neuromorphic overparameterisation and few-shot learning in multilayer physical neural networks. *Nat. Commun.* **15**, 7377 (2024).

60. L. G. Wright, T. Onodera, M. M. Stein, T. Wang, D. T. Schachter, Z. Hu, P. L. McMahon, Deep physical neural networks trained with backpropagation. *Nature* **601**, 549–555 (2022).

61. M. L. Huang, Y. C. Chang, C. H. Chang, Y. J. Lee, P. Chang, J. Kwo, T. B. Wu, M. Hong, Surface passivation of III-V compound semiconductors using atomic-layer-deposition-grown Al2O3. *Appl. Phys. Lett.* **87**, 252104 (2005).

62. Imperial College Research Computing Service, doi:10.14469/hpc/2232.

63. W. F. Schreiber, Image processing for quality improvement. *Proc. IEEE* **66**, 1640–1651 (1978).

64. S. Bandyopadhyay, A. Sludds, S. Krastanov, R. Hamerly, N. Harris, D. Bunandar, M. Streshinsky, M. Hochberg, D. Englund, Single-chip photonic deep neural network with forward-only training. *Nat. Photonics* **18**, 1335–1343 (2024).

65. H. Wang, J. Hu, A. Morandi, A. Nardi, F. Xia, X. Li, R. Savo, Q. Liu, R. Grange, S. Gigan, Large-scale photonic computing with nonlinear disordered media. *Nat. Comput. Sci.* **4**, 429–439 (2024).
